# Transcriptomic and genome-wide association study reveal long noncoding RNAs responding to nitrogen deficiency in maize

**DOI:** 10.1186/s12870-021-02847-4

**Published:** 2021-02-12

**Authors:** Peng Ma, Xiao Zhang, Bowen Luo, Zhen Chen, Xuan He, Haiying Zhang, Binyang Li, Dan Liu, Ling Wu, Shiqiang Gao, Duojiang Gao, Suzhi Zhang, Shibin Gao

**Affiliations:** 1 Maize Research Institute, Sichuan Agricultural University, Chengdu, 611130 Sichuan China; 2 State Key Laboratory of Crop Gene Exploration and Utilization in Southwest China, Chengdu, 611130 Sichuan China

**Keywords:** Maize, Nitrogen use efficiency, RNA-Seq, Genome-wide association study, LncRNA, Co-expression

## Abstract

**Background:**

Long noncoding RNAs (lncRNAs) play important roles in essential biological processes. However, our understanding of lncRNAs as competing endogenous RNAs (ceRNAs) and their responses to nitrogen stress is still limited.

**Results:**

Here, we surveyed the lncRNAs and miRNAs in maize inbred line P178 leaves and roots at the seedling stage under high-nitrogen (HN) and low-nitrogen (LN) conditions using lncRNA-Seq and small RNA-Seq. A total of 894 differentially expressed lncRNAs and 38 different miRNAs were identified. Co-expression analysis found that two lncRNAs and four lncRNA-targets could competitively combine with ZmmiR159 and ZmmiR164, respectively. To dissect the genetic regulatory by which lncRNAs might enable adaptation to limited nitrogen availability, an association mapping panel containing a high-density single–nucleotide polymorphism (SNP) array (56,110 SNPs) combined with variable LN tolerant-related phenotypes obtained from hydroponics was used for a genome-wide association study (GWAS). By combining GWAS and RNA-Seq, 170 differently expressed lncRNAs within the range of significant markers were screened. Moreover, 40 consistently LN-responsive genes including those involved in glutamine biosynthesis and nitrogen acquisition in root were identified. Transient expression assays in *Nicotiana benthamiana* demonstrated that *LNC_002923* could inhabit *ZmmiR159*-guided cleavage of *Zm00001d015521*.

**Conclusions:**

These lncRNAs containing trait-associated significant SNPs could consider to be related to root development and nutrient utilization. Taken together, the results of our study can provide new insights into the potential regulatory roles of lncRNAs in response to LN stress, and give valuable information for further screening of candidates as well as the improvement of maize resistance to LN stress.

**Supplementary Information:**

The online version contains supplementary material available at 10.1186/s12870-021-02847-4.

## Background

Presently, maize (*Zea mays* L.) serves as the major cultivated crop for human consumption and animal feed globally. Nitrogen (N) is an important plant macronutrient and improving N uptake is a key option for increasing crop yield [1]. Nitrate (NO_3_) and ammonium (NH_4_) are the main inorganic nitrogen sources that plant roots take up and assimilate in aerobic soil and flooded soil conditions, respectively [2]. Almost all soils are deficient in N, which directly leads to excessive application of N fertilizer. Therefore, it is more important to reduce the costs of agriculture and its impact on the environment. Recently, researchers found that growth-regulating factor 4 (GRF4) and growth-repressing DELLA proteins act in equilibrium in the regulation of growth and nitrogen metabolism in plants [3], which should help us understand the nitrogen metabolism of plants and contribute to sustainable development of global safe food supply.

Plant nitrate transport proteins include low-nitrate-affinity transporters such as most members of the NRT1 family [4], which are crucial transporters for nitrate uptake and nitrate transportation among cells, tissues, and organs. Meanwhile, high-nitrate-affinity transporter NRT2 families such as *Arabidopsis thaliana* NRT2.5 (AtNRT2.5) [5] are responsible for assimilating nitrate at relatively low concentration ranges. Other nitrate transporters, the chloride channel (CLC) family [6] can mediate nitrate accumulation and transport. Among these protein families, such as *Arabidopsis thaliana* NRT1.1 (AtNRT1.1) [7], which play dual roles as NO3^−^ receptors and transporters. Ammonium transporters are mainly located at the plasma membrane. They are responsible for hydrophobic NH_3_ transport and ammonium distribution, such as *Arabidopsis thaliana* AMT1.1 (AtAMT1.1) [8].

Long noncoding RNAs (lncRNAs) are generally defined as transcripts that cannot encode proteins and have a sequence length ranging from 200 nt to 100 kb. LncRNAs usually regulate adjacent target genes in *cis*, and regulate distant target genes in *trans* [9]. Hence, the positions of lncRNAs are helpful when speculating on their functions. The ceRNA hypothesis, which includes mRNAs, transcribed pseudogenes, and lncRNAs, describes that they can communicate with each other by sharing the same microRNA response elements (MREs) and that RNAs influence each other’s expression through competing miRNAs [10]. For example, the ceRNA linc-RoR was shown to share the same miRNA with core transcription factors (TFs) and prevent these core TFs from miRNA-mediated suppression in self-renewing human embryonic stem cells [11]. Moreover, in maize, the highly abundant Pi-deficiency-induced long-noncoding RNA1 (*PILINCR1*) could efficiently impair the *miR399*-guided cleavage of PHOSPHATE2 (*PHO2*) to regulate *PHO2* level and reduce the tolerance to low Pi [12]. In rice, the potential ceRNA network comprising 376 and 511 lincRNAs of shoot and root, respectively, was identified from RNA-seq data, indicating the systematic regulation of lincRNA function under low Pi stress [13].

Upon completion of the reference genome sequencing of maize inbred line B73, the discovery of single-nucleotide polymorphisms (SNPs) and insertion-deletion polymorphisms lay a foundation for locating QTLs associated with maize agricultural characteristics and exploring candidate genes [14, 15]. Genome-wide association study (GWAS) is based on linkage disequilibrium (LD) to discover the relationship between objective traits and genetic markers in groups [16]. Maize lines presenting natural variation in different parts of the world were studied using GWAS to find the gene *ZmNAC111*, which encodes the transcription factor NAC located on chromosome 10 that plays an important role in maize seedling drought tolerance [17], and 384 maize inbred lines were genotyped with 681,257 SNPs and 22 seedling root architecture traits were applied to identify candidate genes contributing to root development at the seedling stage [18]. In double-haploid maize, a total of 54 SNPs were also identified to be significantly associated with resistance to maize chlorotic mottle virus and lethal necrosis [19]. Furthermore, by combining metabolite profiles and GWAS, five low-Pi-responding consensus genes associated with morphological traits and simultaneously involved in metabolic pathways were mined [20].

Previous researches generally focused on revealing coding genes regulated by nitrogen; in contrast, noncoding components such as lncRNAs induced by nitrogen deficiency have received little attention. In the present study, high-throughput sequencing was thus applied to analyze the expression profiles of lncRNAs, mRNAs, and miRNAs at the maize seedling stage under HN and LN conditions. The target genes of potential lncRNAs and miRNA**–**lncRNA pairs were predicted. We also determined the functions of lncRNAs in the co-expression network based on the “ceRNA hypothesis.” Transient expression assays in *Nicotiana benthamiana* demonstrated that *LNC_002923* could inhibit the cleavage of *Zm00001d015521* by *ZmmiR159*. One hundred and seventy lncRNAs containing significant root trait-associated SNPs were identified. Moreover, a total of 40 consistently LN**–**responsive candidate genes were screened through combining GWAS and RNA-Seq. Together, our results provide multiple insights to understand the LN**–**responsive mechanisms of lncRNAs in maize seedlings and offer new ideas for improving nitrogen use efficiency in maize.

## Results

### High-throughput sequencing of lncRNA and small RNA libraries

**Fig. 1:**
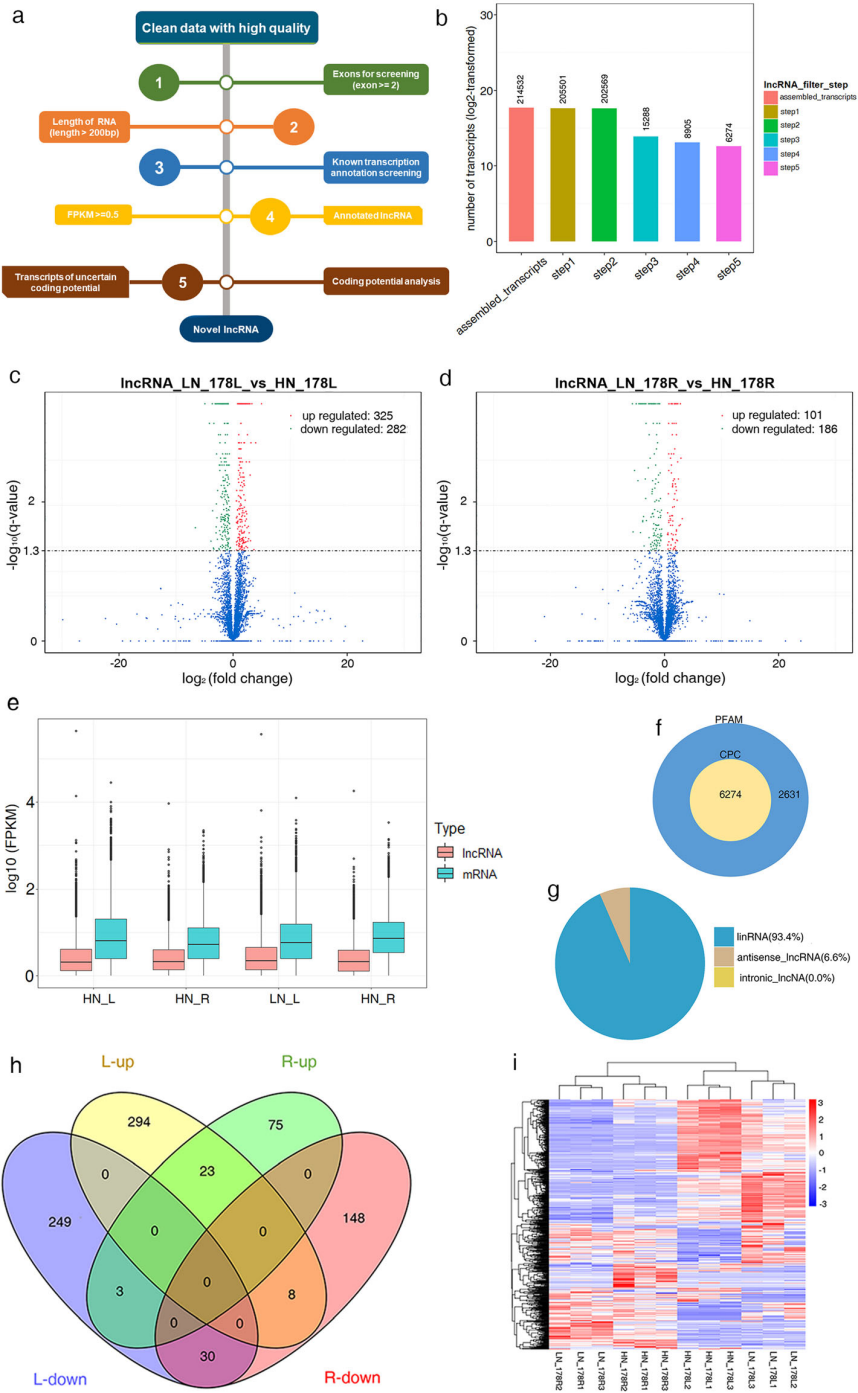
The pipeline used for the identification of lncRNA (**a**). Setting up 5 steps to filter assembled transcripts (**b**). The volcano plot of differential expressed lncRNAs between two nitrogen conditions in leaf (**c**) and (**d**) root. The expression values of lncRNAs and mRNAs were calculated based on the RNA-Seq results, respectively (**e**). LncRNA classification (**f**) and lncRNA coding potential predicted by protein family database (Pfam) and Coding Potential Calculator (CPC) (**g**). Differentially expressed lncRNAs between leaves and roots (**h**). Expression profiles of lncRNA during seedling under HN and LN conditions (**i**). HN, hign nitrogen. LN, low nitrogen. L, leaf. R, root

In total, 1,241,588,130 and 168,839,937 raw reads were generated from the libraries of lncRNAs and small RNAs, respectively. After the removal of low**–**quality reads and trim adapters, we obtained approximately 1,175,852,744 and 165,272,934 clean reads from the lncRNA and small RNA libraries. Then, we mapped the clean reads for each sample to the maize genome (B73 RefGen_V4) and used them for further analysis (Table 1). For mRNAs, a total of 214,532 transcripts were reconstructed from all of the 12 RNA-seq datasets, and 8836 differentially expressed genes were identified in leaves and roots (Fig. S1a, b). For lncRNAs, we based our analysis on the results of transcript splicing and following the structural characteristics and noncoding proteins, setting up a series of strict screening conditions (Fig. 1a). A total of 6274 transcripts were applied to the analysis of differential expression (Fig. 1b). We obtained 894 reliably expressed lncRNAs in leaves and roots (Fig. 1c, d). For miRNAs, the length distribution (range 18–30 nt) of total small RNAs was generated from clean reads (Fig. S2a). The transcripts per million (TPM) of miRNA is shown in Fig. S2b. Then, the screened sRNAs were used to analyze the distribution on the reference sequence and identify known and novel miRNAs. A total of 184 known miRNAs and 106 novel miRNAs were found in leaves and roots (Table S1). The analysis of differential mRNAs and miRNAs expression patterns under HN and LN conditions are presented in Fig. S3.

**Table 1:** Overview of high-throughput sequencing datasets

Sample	Raw reads (mRNA)	Clean reads (mRNA)	Mapped mRNA	Raw reads (sRNA)	Clean reads (sRNA)	Total sRNA	Mapped sRNA
HN_178L1	109,909,236	103,469,546	87,028,986 (84.11%)	13,280,860	12,997,834	10,034,480	9,092,731 (90.61%)
HN_178L2	98,882,294	92,738,214	77,601,590 (83.68%)	13,386,049	13,013,518	11,416,614	10,586,874 (92.73%)
HN_178L3	112,394,988	107,414,484	91,656,287 (85.33%)	14,543,122	14,230,805	11,286,848	10,204,716 (90.41%)
HN_178R1	107,762,888	103,079,810	66,664,905 (64.67%)	14,487,323	14,182,798	10,576,881	3,754,075 (35.49%)
HN_178R2	113,741,976	107,492,062	69,938,707 (65.06%)	12,788,134	12,544,973	9,729,248	3,298,959 (33.91%)
HN_178R3	112,270,478	105,413,706	69,146,376 (65.6%)	14,253,769	13,962,951	9,719,839	3,346,704 (34.43%)
LN_178L1	112,309,946	107,045,930	91,314,837 (85.3%)	14,953,702	14,688,124	12,241,826	11,194,567 (91.45%)
LN_178L2	86,345,420	81,918,980	70,017,448 (85.47%)	14,796,171	14,527,123	8,584,463	7,808,736 (90.96%)
LN_178L3	89,499,196	85,098,370	72,826,546 (85.58%)	13,941,202	13,607,151	10,073,249	9,268,334 (92.01%)
LN_178R1	87,421,586	81,198,490	56,714,397 (69.85%)	13,320,127	13,046,233	9,657,102	5,046,601 (52.26%)
LN_178R2	103,512,304	99,661,226	68,242,687 (68.47%)	14,165,435	13,850,479	10,900,885	5,754,381 (52.79%)
LN_178R3	107,537,818	101,322,926	71,234,057 (70.3%)	14,924,043	14,620,945	10,581,227	5,504,041 (52.02%)

### Genome-wide identification of lncRNAs

In maize seedlings, the lncRNAs ranged in length from 201 base pairs (bp) to approximately 29,176 bp, with a mean length of 829 bp and distributed on each chromosome. The lncRNAs showed lower FPKM than the mRNAs (Fig. 1e). Of these lncRNAs, 93.4% were lincRNAs and 6.6% were antisense lncRNAs (Fig. 1f). Their coding potential was also predicted by PFAM and CPC (Fig. 1g). To identify the lncRNAs involved in responses to nitrogen stress, we selected lncRNAs with a q-value< 0.05 between the HN and LN groups as being differentially expressed. Overall, 607 and 287 differential lncRNAs were genome-widely screened from leaves and roots (Fig. 1c, d), respectively, of which 23 were consistently upregulated and 30 were consistently downregulated in both roots and leaves (Fig. 1h). This suggested that these lncRNAs perform similar functions in different tissues in response to LN stress. The clustering analysis of different lncRNAs was applied to determine the cluster model under HN and LN conditions (Fig. 1i); it showed upregulated and downregulated lncRNAs in the two tissues. Besides, 477 differentially expressed transcripts of uncertain coding potential (TUCPs) potentially containing a subset of lncRNAs with certain coding potential were identified (Fig. S1c, d). The characteristics of identified lncRNAs are shown in Fig. S4. In these results, we obtained a majority of lincRNAs and antisense lncRNAs, and a low proportion of TUCPs, which indicated that the RNA-Seq strategy can be used for these RNAs.

**Fig. 2:**
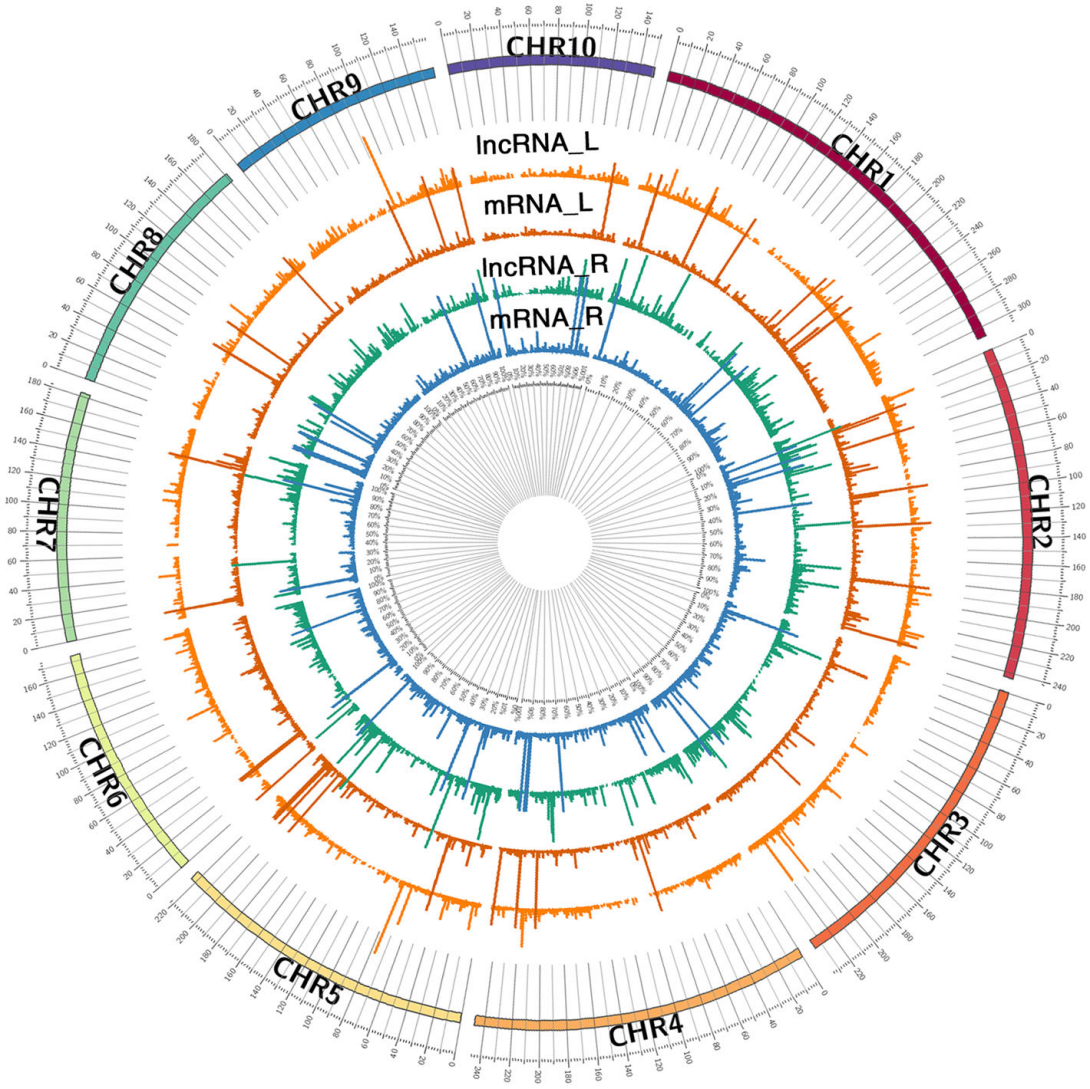
The distribution of lncRNAs and mRNAs on 10 chromosomes. The abundance level of lncRNAs and mRNAs (log10 (fold change)) on each chromosome in leaves and roots

The expressional level mRNA distribution from the 12 libraries is shown along 10 chromosomes (Fig. 2). To understand the potential interaction between lncRNAs and coding transcripts, we identified the adjacent genes within 100 kb either up and downstream from the lncRNAs (Table S2). We also used Pearson’s correlation coefficient to analyze the correlation of expression level between lncRNAs and genes; those with a correlation value greater than 0.95 were taken for analysis (Table S2). The target analysis of co-location and co-expression provides an approach to predict the main functions of lncRNAs.

### Validation of lncRNA, lncRNA target, and miRNA expression using qRT-PCR

**Fig. 3:**
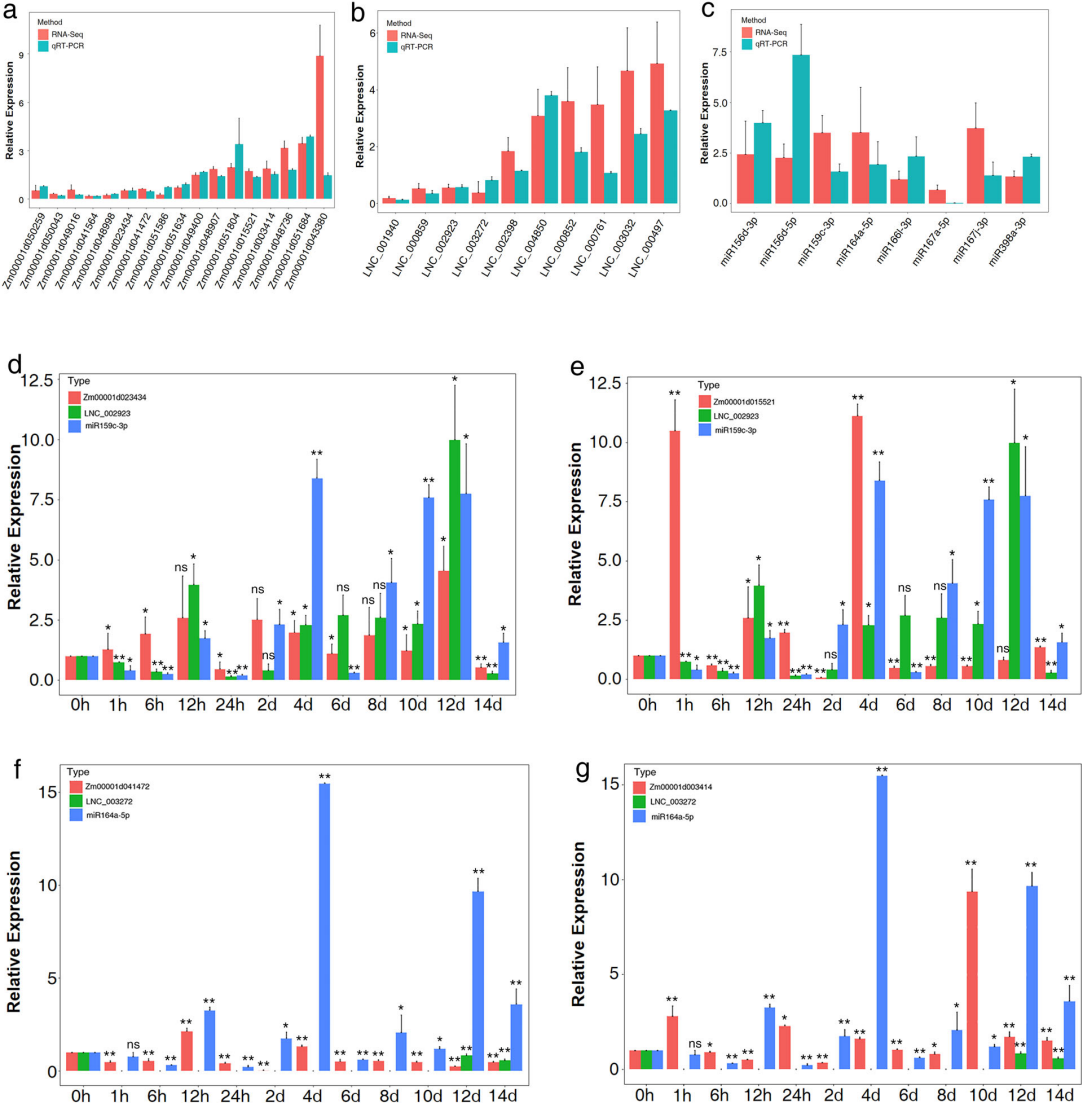
qRT-PCR was performed to validate the differentially expressed mRNAs (**a**), lncRNAs (**b**) and miRNAs (**c**) identified by the RNA-seq results. There was no significant difference between RNA-Seq and qRT-PCR. qRT-PCR was performed to analysis the dynamic response of lncRNA-miRNA-mRNA expression levels to LN stress at different treatment stages.*, significant at *P* < 0.05. **, significant at *P* < 0.01. ns, not significant were relative to 0 h

To confirm the reliability of the deep sequencing results, 17 mRNAs, 10 lncRNAs, and 8 miRNAs were randomly selected for qRT-PCR analysis. As shown in Fig. 3a–c, the qRT-PCR, and RNA-seq data showed the same tendency, indicating the reliability of the RNA-seq results.

### Analysis of the co-expression network

**Fig. 4:**
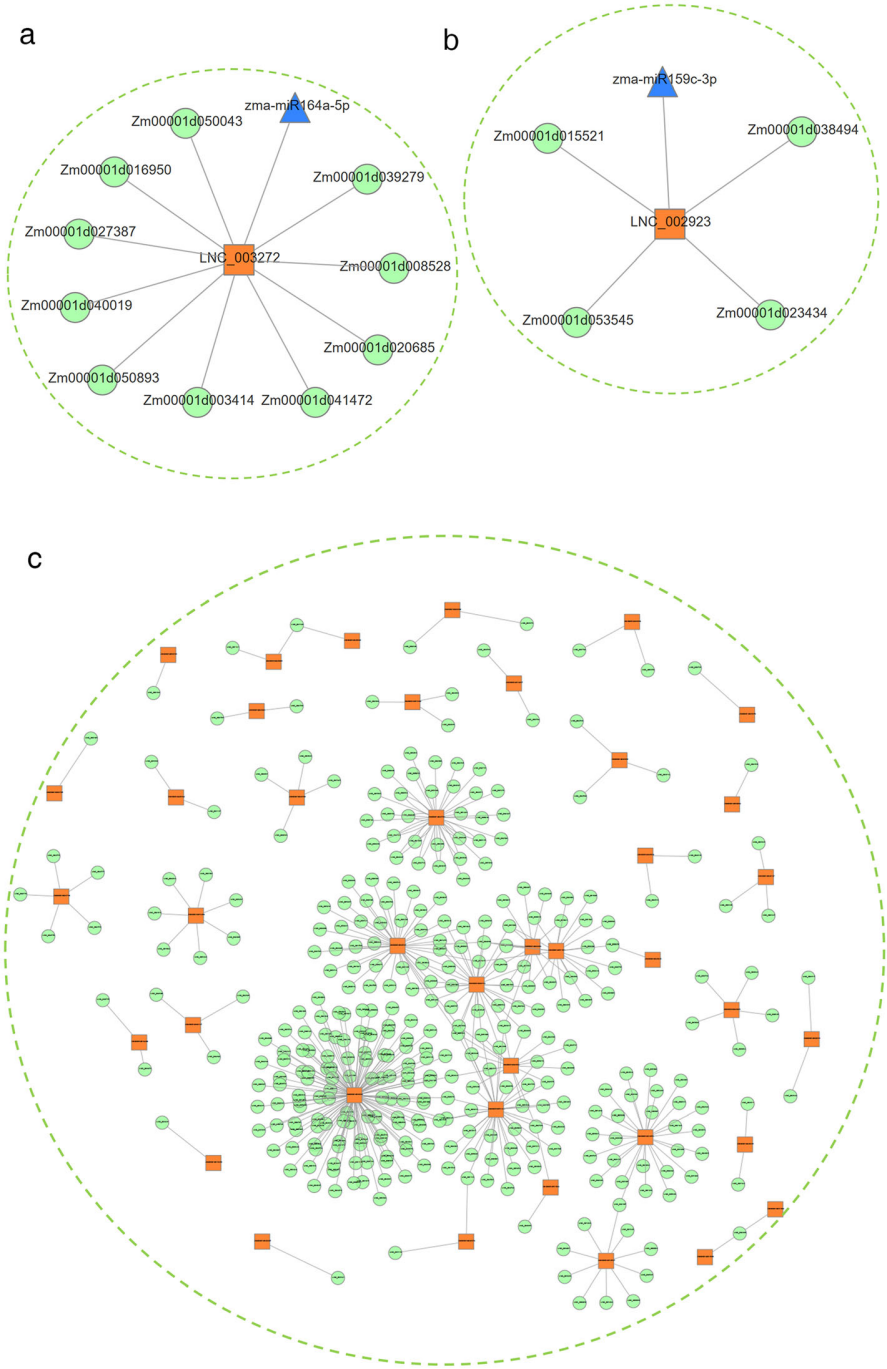
The lncRNA-gene pairs which shared same miRNA binding sites with miRNA, root- (**a**) and leaf- (**b**) special co-expression networks. The 40 consistently candidate genes were influenced by lncRNAs in *cis* and *trans* (**c**). Green nodes represent lncRNAs, orange nodes represent mRNAs and blue triangles represent miRNAs

A total of 38 differentially expressed miRNAs identified from 12 small RNA libraries (Fig. S1e, f) were utilized for analyses of their interactions with lncRNAs and mRNAs. And then, we constructed the co-expression network that lncRNA**–**gene pairs generally share the same miRNA binding sites [10]. As shown in Fig. 4a, b, 2 lncRNAs, 2 miRNAs, and 14 mRNAs were included in the two co-expression networks, and the lncRNAs served as ceRNAs to communicate with many mRNAs through competing with specific miRNAs. These results contributed to our understanding the potential functions of lncRNAs in maize seedling under LN stress, and revealed the mechanism of lncRNAs regulated gene expression at the whole transcriptome.

We then analyzed the dynamic responses of lncRNA–miRNA–mRNA expression levels to LN stress at different treatment stages. For the co-expression network in the leaf, *LNC_002923*, *miR159c*, and two mRNAs were selected for qRT-PCR. As shown in Fig. 3d, e, the expression profile of *Zm00001d023434* was similar to *LNC_002923*, but opposite of miR159c. Furthermore, *Zm00001d015521* in the lncRNA-associated co-expression network showed the opposite expression pattern to *LNC_002923*, but a similar one to *miR159c* at 14 days. In general, the level of *Zm00001d023434* was upregulated under the nitrogen starvation of seedling leaves, except for at 24 h and 14 days. *Zm00001d015521* was almost completely suppressed at most stages, except for 1 h, 12 h, 24 h, and 4 days. The level of *miR159c* was upregulated in leaf at most treatment stages, only showed downregulation at 1 h, 6 h, 24 h, and 6 days. The level of *LNC_002923* was almost always downregulated in the short**–**term, but was upregulated in the long**–**term. For the co-expression network in the root, the expression of *LNC_003272*, *miR164a*, and two mRNAs is shown in Fig. 3f, g. The expression of *LNC_003272* was hardly detected, only showing downregulation at 12 and 14 days. The levels of *miR164a* and mRNAs showed the opposite expression pattern at 14 days after LN stress. Overall, the expression profiles of nitrogen-responsive lncRNAs, miRNAs, and mRNAs match better at a later stage than other short**–**term treatment for the “ceRNA hypothesis”, suggesting that ceRNA mechanisms are vital for further studying lncRNA functions under LN condition in maize.

### Targets analysis of LN-responsive lncRNAs

To detect the *cis*-acting lncRNAs function, we screened 100 kb upstream and downstream of the 607 and 287 differentially expressed lncRNAs in leaves and roots, respectively, and performed lncRNA-mRNA pairs expression correlation analysis. In total, 2561 and 1142 lncRNA-mRNA pairs for differentially expressed lncRNAs in leavesand roots were found, respectively (Table S3). GO analysis predicted some differential genes in the following subcategories within the main category of the biological process: nitrogen compound metabolic process, response to oxidative stress, and chromatin remodeling. Besides, there were also enriched GO terms such as nitrogen compound transport, organ nitrogen compound biosynthetic process, nitrate metabolic process, and regulation of external response (Table S5).

On the other hand, to detect the effect of trans-acting lncRNAs on genes expression regulation in diverse biological processes. According to the expression correlation between lncRNAs and mRNAs (Pearson correlation> 0.95), 59,577 and 22,227 co-expression relationships of lncRNAs and LN-responsive genes were found in leaf and root, respectively (Table S4). GO categories and subcategories were analyzed, the results predicted that most of these genes were organic substance biosynthetic, alpha-amino acid metabolic, and photosynthetic membrane in leaf and root.

We next analyzed the KEGG pathway enrichment analysis with differentially expressed genes, and the top20 enriched pathways are presented in (Table S6). The results showed the trans-acting lncRNAs were enriched in several pathways associated with cysteine and methionine metabolism, cyanamino acid metabolism. Meanwhile, some enriched pathways associated with nitrogen metabolism, alanine, aspartate, and glutamate metabolism were identified. In *cis*-acting, the results of these genes function in the chlorophyll metabolism, biosynthesis of secondary metabolites, as well as carbon fixation in photosynthetic organisms. These enriched biological processes and pathways are related to glutamine family amino acid biosynthetic process and abiotic stress, indicating that the differentially expressed LN-responsive lncRNAs play important roles in the absorption and transportation of nitrogen during maize development.

### *LNC_002923* inhibits the cleavage of *Zm00001d015521* by *ZmmiR159*

**Fig. 5:**
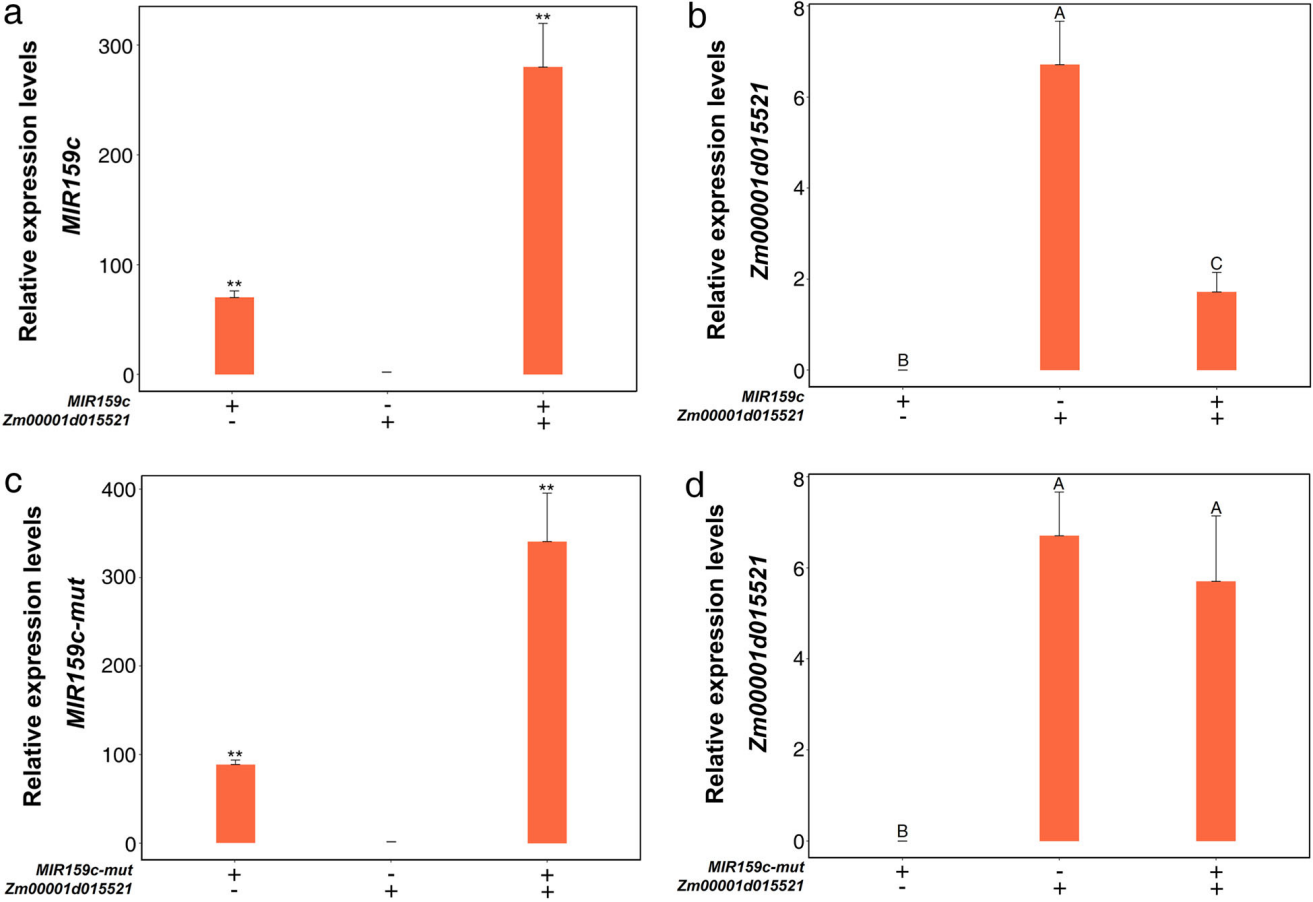
Co-expression the combination expression plasmid of *MIR159c/Zm00001d015521* (**a**, **b**) and *MIR159c-mut/Zm00001d015521* (**c**, **d**) in *N. benthamiana*. The relative expression levels of *MIR159c* (**a**) and *MIR159c-mut* (**c**) were detected by qRT-PCR, and the data were normalized using U6 gene. The relative expression level of *Zm00001d015521* (**b**, **d**) were normalized using the 18S gene of tobacco. Same letters are not significant at *P* < 0.01. **, significant at *P* < 0.01

**Fig. 6:**
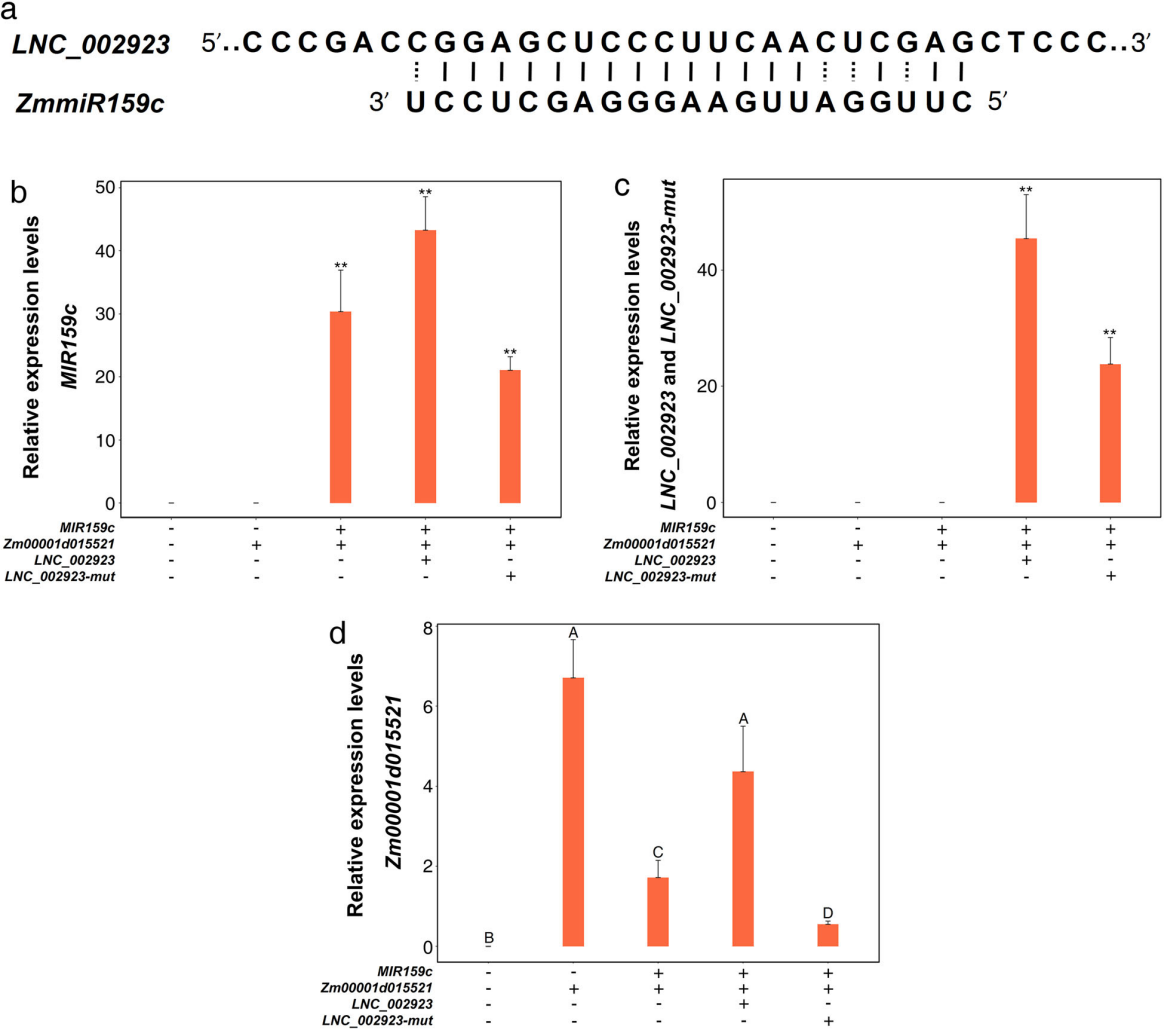
The diagram of complementary binding sequence of *LNC_002923* and *ZmmiR159* in maize (**a**). Co-expression the combination expression plasmid of *MIR159c*, *Zm00001d015521*, *LNC_002923*, *LNC002923-mut* (**b**, **c**, **d**). The relative expression level of *MIR159c* (**b**) were normalized using U6 gene. The relative expression levels of mRNA and lncRNA were normalized using the 18S gene of tobacco (**c**, **d**). Same letters are not significant at *P* < 0.01. **, significant at *P* < 0.01

According to our previously predicted co-expression regulatory networks, experiments were conducted to verify whether lncRNA affected the mRNA-miRNA pair. The *Zm00001d015521* was the predicted target gene of *ZmmiR159c*. We used *ZmmiR159c* and *ZmmiR159c-mut* to construct the transient expression assay in *Nicotiana benthamiana*, in which *ZmmiR159c-mut* was designed primers for six loci in *ZmmiR159c* mature sequence to introduce the target mutation. The expression levels of *ZmmiR159c* and *ZmmiR159c-mut* were detected by qRT-PCR (Fig. 5a, c). As shown in Fig. 5b, d, the expression level of *Zm00001d015521* significantly decreased when co-expressed with *ZmmiR159c*, however, the expression level of *Zm00001d015521* was not affected when coexpressed with *ZmmiR159c-mut*. These results indicate that *Zm00001d015521* is one of the target genes of ZmmiR159c. Previous studies have found that lncRNA could bind to miRNA, thus relieving the inhibitory effect of miRNA on target genes. In this study, the binding site of *ZmmiR159c* was found in *LNC_002923* sequence (Fig. 6a). We also introduced six mutations in the *ZmmiR159c* sequence of *LNC_002923 (LNC_002923-mut)*. Then, we conducted transient expression assays in *Nicotiana benthamiana* to detect whether *LNC_002923* could inhabit the cleavage of *Zm00001d015521* by *ZmmiR159c*. The expression levels of *ZmmiR159c, LNC_002923* and *LNC_002923-mut* were detected by qRT-PCR (Fig. 6b, c). As shown in Fig. 6d, the expression level of *Zm00001d015521* was not affected when *LNC_002923* was co-expressed with *ZmmiR159c*, indicating that *LNC_002923* could effectively inhibit the cleavage of *Zm00001d015521* by *ZmmiR159c*. However, the *Zm00001d015521* expression level was significantly decreased when co-expressed with *LNC_002923-mut*. These results suggested that *LNC_002923* could reduce the inhibitory effect of *ZmmiR159c*.

### Phenotypic differences under HN and LN conditions

The descriptive statistics and broad heritability estimates (H^2^) of 17 maize seedling traits in HN and LN are presented in Table 2. The significance of genotypes and contrasting N concentrations on maize seedlings could be revealed by the analysis of variance results. Most traits showed significant differences between LN stress and the control, except the seminal root number (SRN). These findings indicated that collection of natural populations was sufficiently diverse for the subsequent association analysis. On average, shoot growth was limited under LN condition, while root growth was enhanced. The shoot length (SL) was greater under HN (36.278) than under LN (30.377) treatment. Moreover, root dry weight (RDW) and total root length (TRL) were higher under LN than under HN. Pearson’s correlation coefficients were calculated for the 17 collected traits under contrasting nitrogen levels listed in Table S8.

**Table 2:** Statistics of 17 traits collected

Trait	Treatment	Mean	Sd	Min	Max	Skew	Kurtosis	H^**2**^	F-value (Treatment)	F-value (Genotype)
SL (cm)	HN LN	36.278 30.377	6.113 5.367	17.400 16.567	52.367 48.233	0.152 0.138	0.124 0.026	0.580 0.570	265**	5.136** 5.028**
LN	HN LN	3.557 3.370	0.363 0.357	2.700 2.300	5.100 5.000	0.252 0.471	0.497 1.638	0.415 0.404	87.15**	3.128** 3.038**
PRL (cm)	HN LN	17.968 16.758	3.927 3.834	4.567 7.250	28.300 33.567	0.181 0.280	0.003 0.782	0.271 0.279	21.34**	2.135** 2.171**
CRN	HN LN	4.030 4.104	1.001 0.957	0.000 1.000	8.000 6.667	0.111 0.054	1.911 0.051	0.410 0.397	1.498**	3.03** 2.978**
SRN	HN LN	6.332 6.415	1.842 1.803	2.333 2.556	13.667 12.667	0.716 0.525	1.045 0.186	0.677 0.660	1.216^ns^	7.299** 6.829**
RFW (g)	HN LN	0.793 0.690	0.221 0.183	0.273 0.298	1.518 1.397	0.467 0.563	0.122 0.438	0.621 0.610	112.7**	5.914** 5.744**
SFW (g)	HN LN	1.375 0.955	0.405 0.262	0.450 0.442	2.551 1.850	0.271 0.417	0.256 0.247	0.552 0.577	451.7**	4.703** 5.057**
TPB (g)	HN LN	0.057 0.061	0.014 0.015	0.028 0.033	0.109 0.115	0.637 0.602	1.291 0.544	0.638 0.649	31.81**	6.311** 6.548**
LDW (g)	HN LN	0.106 0.085	0.030 0.024	0.045 0.043	0.213 0.195	0.463 0.762	0.131 1.072	0.585 0.593	180.5**	5.314** 5.562**
TDW (g)	HN LN	0.163 0.147	0.041 0.036	0.074 0.083	0.302 0.296	0.565 0.776	0.607 0.945	0.609 0.622	67.59**	5.753** 5.99**
TRL (cm)	HN LN	267.097 268.077	92.748 100.252	35.040 71.685	649.066 632.929	0.534 0.515	0.491 0.134	0.472 0.516	0.18**	3.686** 4.196**
SA (cm^2^)	HN LN	49.225 45.211	15.109 14.607	12.737 16.316	103.775 100.769	0.491 0.634	0.201 0.537	0.545 0.562	29.8**	4.586** 4.845**
ARD (cm)	HN LN	0.629 0.585	0.079 0.075	0.424 0.399	1.002 0.871	0.474 0.472	1.416 0.871	0.571 0.640	107.5**	5.225** 6.79**
RV (cm^3^)	HN LN	0.756 0.632	0.247 0.199	0.290 0.287	1.652 1.227	0.820 0.819	0.696 0.377	0.609 0.616	130.1**	5.681** 5.803**
Tips	HN LN	337.396 345.986	133.261 136.940	51.667 96.750	756.667 852.800	0.600 0.566	0.080 0.171	0.516 0.571	0.614**	4.197** 4.997**
Forks	HN LN	1043.947 1057.359	470.938 535.802	117.000 249.857	2939.571 3568.400	0.807 1.145	0.655 2.286	0.618 0.633	0.071**	5.853** 6.182**
Crossings	HN LN	132.270 147.887	83.802 95.869	9.333 13.833	477.750 607.600	1.114 1.278	1.104 2.347	0.495 0.518	6.616**	3.942** 4.219**

**Significant at ***P*** **< 0.01;** ***ns*** **Not significant;** ***H***^***2***^ **Broad-sense heritability;** ***HN*** **High nitrogen;** ***LN*** **Low nitrogen.** ***SL*** **Shoot length;** ***LN*** **Leaf number;** ***PRL*** **Primary root length;** ***CRN*** **Crown root number;** ***SRN*** **Seminal root number;** ***RFW*** **Root fresh weight;** ***SFW*** **Shoot fresh weight;** ***TPB*** **Total plant biomass;** ***LDW*** **Leaf dry weight;** ***TDW*** **Total dry weight;** ***TRL*** **Total root length;** ***SA*** **Surface area;** ***ARD*** **Average root diameter;** ***RV*** **Root volume**

### Candidate genes revealed by genome-wide association analysis

For the genome-wide identification of significant SNPs associated with traits, the software TASSEL 5.0 was selected to conduct GWAS with 46,108 high-quality SNPs (MAF > 0.01) using a mixed linear model (MLM) and markers with a *p*-value of <1e− 4.6 were considered for candidate gene analysis. A total of 23 significant markers were detected by low nitrogen tolerance index (LNTI) and three markers were detected under LN treatment. Among them, 12 SNPs were found to be associated with the crown root number (CRN). Seven and three significant associations corresponded to Forks and Tips, respectively, and three markers were associated with each of Crossings and average root diameter (ARD). Moreover, one SNP was found to be significantly associated with two root traits (Forks and Tips). According to the average linkage disequilibrium (calculated by TASSEL 5.0) decay distance across all 10 maize chromosomes within the mapping population, a total of 1474 candidate genes were screened near these significantly associated SNP markers. Three significant markers were associated with 232 genes under LN treatment, namely, PUT-163a-13,126,581-167, PZE-104092320, and PZE-109052750, and they were respectively associated with seminal root number (SRN), Crossings, and Tips and located on chromosomes 4, 6, and 9. Meanwhile, 23 significant SNPs associated with 1242 candidate genes were revealed by LNTI value. A list of all significant markers’ trait associations is presented in Table S7. According to the location of identified lncRNAs, a total of 36 and 134 differently expressed lncRNAs within the range of significant markers were screened under LN and LNTI, respectively (Table S7). These LN-responsive candidate genes and lncRNAs could play vital roles in regulating root development during the maize seedling stage.

### Combining GWAS and expression profile to mine consistent candidate genes

**Fig. 7:**
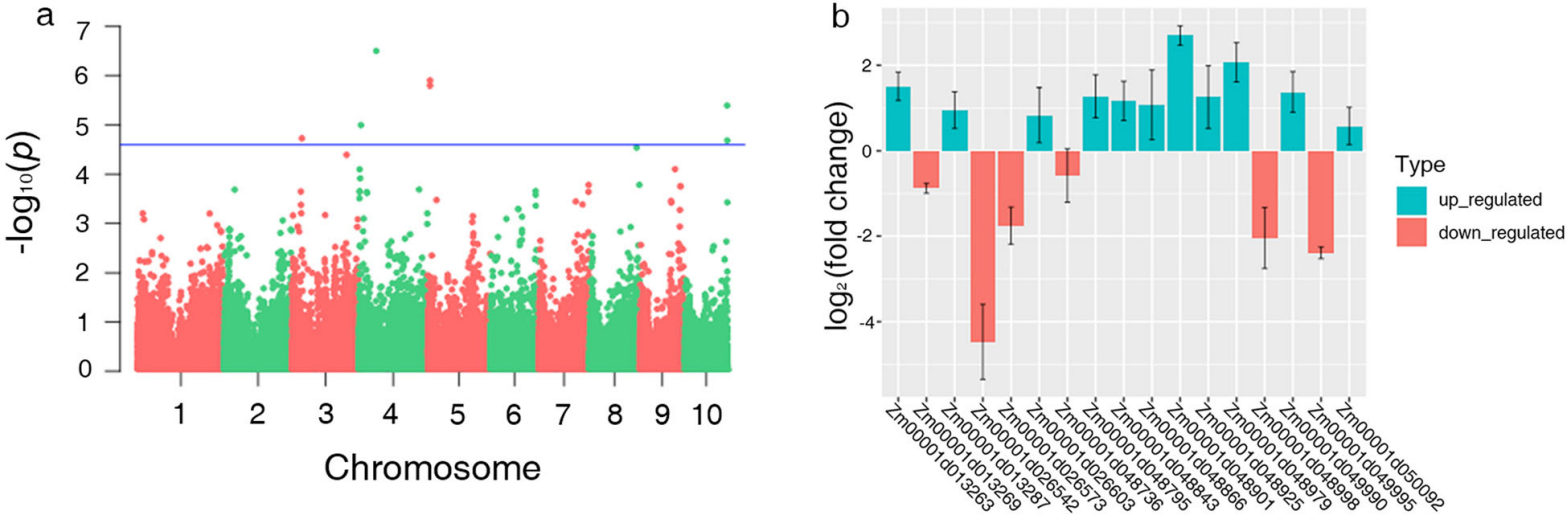
Manhattan plots of the MLM for FORKs in lines (**a**). Transcript level difference of candidate genes detected in RNA-Seq of Log2 (fold change) of FPKM around significant SNPs

Combined with GWAS and RNA-Seq, we found that among 232 candidate genes detected by GWAS under LN condition, 16 and 29 candidate genes respectively showed obvious downregulation and upregulation patterns, and 7 candidate genes were expressed in roots and leaves (Fig. S6a and Table S7). At the same time, among 1013 candidate genes detected by GWAS under LNTI, 107 and 125 candidate genes showed downregulation and upregulation respectively, among which 38 candidate genes were expressed in roots and leaves (Fig. S6b and Table S7). Besides, integrating the results of multiple RNA-seq and GWAS, a total of 10 significant SNPs (*P* < 1e− 4.6) were associated with 34 candidate genes identified by using LNTI of root traits, and one significant SNP associated with six candidate genes was identified under LN stress (Table 3). The expression levels of candidate genes in which GWAS-identified SNPs located were evaluated in 255 maize lines through RNA-Seq of root at the maize seedling stage under LN stress. This includes the discovery of two genes in the integration of meta-analysis and large-scale gene expression profile data of maize under LN stress [22]. For example, *Zm00001d051804* plays an important role in glutamine biosynthesis in response to exogenous nitrogen during seed germination in maize and Arabidopsis [26]. In addition, *Zm00001d048998* is a chlorophyll A-B binding protein, which is involved in the absorption and utilization of nitrogen nutrients in maize [﻿^21^﻿]. For other genes, *Zm00001d051666* and *Zm00001d049380* were found to be associated with nitrogen acquisition in maize roots [23]. Finally, six candidate genes were found in RNA-seq of maize root in response to nitrogen stress [24], and 29 genes were consistently detected upon comparing with research involving GWAS of carbon and nitrogen metabolism in maize [25], respectively. The Manhattan plots for Forks and fold change values from RNA-Seq are shown in Fig. 7a, b. These consistent candidate genes explored in our research could contribute strongly to deficient nitrogen tolerance.

**Table 3:** Results for SNP loci with root traits and consistent candidate genes jointly identified by association and RNA-Seq

									Candidate genes based on RNA-Seq^c^	
Trait	SNP	Chr	Position^a^	P	Alleles	R^2^ (%)	Gene^b^	Reference	Annotation	Fold change
Forks	PZE-104010552	4	7,929,588	5.0 × 10^−5^	A/G	8.9	*Zm00001d048998*	Mu et al. 2017 [21]	Chlorophyll a-b binding protein CP26	−2.03939
Crossings	PZE-104092320	4	167,437,768	4.7 × 10^−5^	A/G	1.8	*Zm00001d051804*	Luo et al. 2015 [22]	Glutamine synthetase root isozyme 4	1.65671
Forks	PZE-104043052	4	61,411,237	6.5 × 10^− 5^	A/C	11	*Zm00001d049995*		Nitrate reductase [NADH]	−2.39002
Crossings	PZE-104092320	4	167,437,768	4.7 × 10^−5^	A/G	1.8	*Zm00001d051666*	Zanin et al. 2015 [23]	Neutral/alkaline invertase	1.83246
PRL	ZM005894–0463	4	31,060,531	5.6 × 10^−5^	A/C	9.9	*Zm00001d049380*		Putative aminotransferase superfamily protein	0.66934
CRN	PZE-104016717	4	16,500,659	5.2 × 10^−5^	A/G	11	*Zm00001d049059*	He et al. 2016 [24]	Alcohol dehydrogenase2	0.766936
Tips	PZE-104049074	4	75,505,829	5.2 × 10^−5^	A/G	8.7	*Zm00001d050195*		WRKY DNA-binding domain protein	−0.795948
Forks	PZE-104010552	4	7,929,588	5.0 × 10^−5^	A/G	8.9	*Zm00001d048866*		(Z)-3-hexen-1-ol acetyltransferase	1.07489
Forks	PZE-104010552	4	7,929,588	5.0 × 10^−5^	A/G	8.9	*Zm00001d048736*		S-alkyl-thiohydroximate lyase SUR1	−0.576864
CRN	PZE-105047147	5	36,470,371	6.1 × 10^−5^	A/G	13	*Zm00001d014200*		Expressed protein	0.402627
Forks	PUT-163a-91,875,212-4781	5	7,724,698	5.9 × 10^−5^	A/G	10	*Zm00001d013263*		Expressed protein	1.51149
Forks	PZE-104043052	4	61,411,237	6.5 × 10^−5^	A/C	11	*Zm00001d050092*	Zhang et al. 2015 [25]	Putative subtilase family protein	0.585778
Forks	PZE-104043052	4	61,411,237	6.5 × 10^−5^	A/C	11	*Zm00001d049990*		Putative ENTH/ANTH/VHS protein	1.38056
PRL	ZM005894–0463	4	31,060,531	5.6 × 10^−5^	A/C	9.9	*Zm00001d049554*		Putative glycerol-3-phosphate transporter 1	−0.807844
PRL	ZM005894–0463	4	31,060,531	5.6 × 10^−5^	A/C	9.9	*Zm00001d049551*		Putative cytochrome P450 protein	2.50626
PRL	ZM005894–0463	4	31,060,531	5.6 × 10^−5^	A/C	9.9	*Zm00001d049502*		maternal effect embryo arrest 60	0.542646
PRL	ZM005894–0463	4	31,060,531	5.6 × 10^−5^	A/C	9.9	*Zm00001d049464*		B-box type zinc finger family protein	0.980937
PRL	ZM005894–0463	4	31,060,531	5.6 × 10^−5^	A/C	9.9	*Zm00001d049418*		Germin-like protein subfamily 1 member 8	1.11388
PRL	ZM005894–0463	4	31,060,531	5.6 × 10^−5^	A/C	9.9	*Zm00001d049407*		Polyphenol oxidase chloroplastic	−0.61096
PRL	ZM005894–0463	4	31,060,531	5.6 × 10^−5^	A/C	9.9	*Zm00001d049365*		Methionine aminopeptidase	0.319478
CRN	PZE-104016717	4	16,500,659	5.2 × 10^−5^	A/G	11	*Zm00001d049187*		Phosphogluconate dehydrogenase3	−1.54203
CRN	PZE-104016717	4	16,500,659	5.2 × 10^−5^	A/G	11	*Zm00001d049158*		Transcription factor bHLH62	1.27949
CRN	PZE-104016717	4	16,500,659	5.2 × 10^−5^	A/G	11	*Zm00001d049127*		Putative serine/threonine protein	−0.848596
Forks	PZE-104010552	4	7,929,588	5.0 × 10^−5^	A/G	8.9	*Zm00001d048979*		Putative sucrose-phosphate synthase protein	2.06952
Forks	PZE-104010552	4	7,929,588	5.0 × 10^−5^	A/G	8.9	*Zm00001d048925*		Hydroquinone glucosyltransferase	1.25909
Forks	PZE-104010552	4	7,929,588	5.0 × 10^−5^	A/G	8.9	*Zm00001d048901*		Transcription factor bHLH47	2.69849
Forks	PZE-104010552	4	7,929,588	5.0 × 10^−5^	A/G	8.9	*Zm00001d048843*		Math-btb9	1.16984
Forks	PZE-104010552	4	7,929,588	5.0 × 10^−5^	A/G	8.9	*Zm00001d048795*		SPR1	1.27812
Forks	PZE-104010552	4	7,929,588	5.0 × 10^−5^	A/G	8.9	*Zm00001d048709*		benzoxazinless1	−5.53653
Forks	PZE-110109365	10	148,503,798	4.7 × 10^−5^	A/G	7.5	*Zm00001d026605*		Endoglucanase 5	−5.62458
Forks	PZE-110108403	10	148,106,661	5.4 × 10^−5^	A/C	8.9	*Zm00001d026603*		Mg chelatase subunit H 1	0.836012
Forks	PZE-110108403	10	148,106,661	5.4 × 10^−5^	A/C	8.9	*Zm00001d026573*		Methylthioribose kinase	−1.75366
Forks	PZE-110108403	10	148,106,661	5.4 × 10^−5^	A/C	8.9	*Zm00001d026542*		Myb family transcription factor PHL5	−4.47132
CRN	PZE-105047221	5	36,532,569	5.6 × 10^−5^	A/G	11	*Zm00001d014555*		Tyrosine-sulfated glycopeptide receptor 1	−1.98805
Forks	PUT-163a-91,875,212-4781	5	7,724,698	5.9 × 10^−5^	A/G	10	*Zm00001d013287*		–	0.957732
Forks	PUT-163a-91,875,212-4781	5	7,724,698	5.9 × 10^−5^	A/G	10	*Zm00001d013269*		Beta-glucanase3	−0.88007
Crossings	PZE-104092320	4	167,437,768	4.7 × 10^−5^	A/G	1.8	*Zm00001d051829*		U-box domain-containing protein 44	0.605786
Crossings	PZE-104092320	4	167,437,768	4.7 × 10^−5^	A/G	1.8	*Zm00001d051692*		Aspartyl protease AED1	0.609318
Crossings	PZE-104092320	4	167,437,768	4.7 × 10^−5^	A/G	1.8	*Zm00001d051664*		2OG and Fe (II)-dependent superfamily protein	0.924731
Crossings	PZE-104092320	4	167,437,768	4.7 × 10^−5^	A/G	1.8	*Zm00001d051598*		Plant-specific domain TIGR01570 protein	1.82658

^**a**^ **The physical position of significant SNPs were based on the Maize B73 RefGen_v3.** ^**b**^ **The Genes were screened near these significantly associated SNP markers.** ^**c**^ **The candidate genes were identified by combining with RNA-Seq and previous studies, the expression (Log**_**2**_**(fold change)) based on us RNA-Seq**

To investigate the regulatory roles of lncRNAs in maize seedlings under LN stress, using the predictive lncRNA targets, potential lncRNAs that could influence 40 consistently LN-response candidate genes in a *cis* or *trans* manner were identified. As shown in Fig. 4c, a total of 354 lncRNAs were found. Furthermore, these targets of lncRNAs contained significant SNPs for multiple root traits (Table S9), and lncRNA targets were found to be significantly associated with nitrogen metabolism and the abiotic stress response pathway. Of these lncRNAs, we found LNC_002984, LNC_002985, and LNC_002986 located upstream of *Zm00001d051804* that could potentially regulate the expression levels of neighboring genes in a *cis* manner. *Zm00001d051804* was reported to be related to the response to low nitrogen stress [22]. Moreover, the homologous gene in Arabidopsis was shown to play an important redundant role in ammonium assimilation under ammonium-deficient conditions and in facilitating nitrogen remobilization in root [27, 28]. This implies these lncRNAs could play an important role in root development, especially in nitrogen absorption, transfer, and assimilation in plants. The present analysis provides new information for understanding lncRNAs as important regulators in growth and development associated with LN stress.

## Discussion

Increasingly researches have focused on the regulatory mechanisms of noncoding RNA. Many potential lncRNAs were identified in mammals and plants, and were found to be associated with human diseases and regulation of the expression levels of genes involved in biological processes in plants [29–31]. The ceRNA hypothesis is a new option for explaining how lncRNAs and protein-coding genes communicate with each other through microRNA response elements (MREs). Genome-wide screening and analysis of lncRNAs can provide new insights into how plants respond to LN stress. In the present study, deep sequencing of 12 lncRNA and 12 small RNA libraries created from maize seedlings at the early stages of exposure to HN and LN conditions was performed. We identified 894 reliably differentially expressed lncRNAs and 38 miRNAs, including 19 novel miRNAs.

### MicroRNAs mediate communication between lncRNAs and mRNAs

MicroRNAs as the core of the co-expression network are responsible for mediating lncRNAs and mRNAs. We identified *miR159c* and *miR164a* could communicate with individual lncRNAs, and these lncRNAs were communicated by competing specific miRNAs. *MiR159* was shown to control the expression of GAMYB-like genes in anthers and seeds; these transcription factors are involved in GA-induced aleurone development and death [32], and was also reported to regulate many target genes, such as MYB transcription factors and conserved R2R3 domain [33]. The inhibition induced by *miR159* could reduce the repression of MYB33/65 and play an important role in vegetative development [34]. Specifically, *miR159* has been reported to participate in response to phosphate starvation [35]. However, studies on *miR159* functions in the context of LN stress have not been reported. *MiR164* was reported to participate in lateral root initiation [36] and LN stress response [37]. Under nitrate-starvation condition, *miR164* could cut its target gene NAC1 between the 10th and 11th bases at the post-transcriptional level to resist LN availability [38]. In conclusion, under the co-expression networks, *LNC_003272* and *LNC_002923* could play pivotal roles in biological processes, including root system development and seed germination, as well as potentially participating in inhibiting the effects of miRNAs in response to nitrogen deficiency.

### The co-expression network showed dynamic expression and regulation patterns during the seedling processes

According to the results, there were 2 lncRNAs, 2 miRNAs, and 14 mRNAs included in the network, forming two co-expression regulatory networks (Fig. 4a, b). The analysis of qRT-PCR showed the pattern of co-expression networks similar to the ceRNA hypothesis at most stages after LN treatment. For the co-expression network in leaf, the expression trend of *Zm00001d023434* was consistent with that of *LNC_002923*, but opposite to that of miR159c. In the root, the expression of miR164a showed the opposite pattern to *Zm00001d041472*, but was consistent with that of *Zm00001d003414* at 14 days after LN stress. The expression of *LNC_003272* could not be detected at most stages, except 10 and 14 days. Meanwhile, the gene *Zm00001d015521* was reported to regulate the metabolism of the plant hormone cytokinin and to be influenced by chlorophyll biosynthesis genes, and increased the number of absorbing roots and promoted the growth of seedlings [39]. Moreover, *Zm00001d041472* was found to participate in nitrogen accumulation in vegetable tissue and improve stay-green and yields in maize [40]. Taking these findings together, we suggested *LNC_002923* and *LNC_003272* could participate in the pathway of nitrogen utilization to regulate chloroplast development, nitrogen utilization, as well as potentially participating in inhibiting the effects of miRNAs in response to nitrogen deficiency.

### *LNC_002923* inhibits the cleavage of *Zm00001d015521* by *ZmmiR159*

Previous studies indicated that lncRNAs could regulate mRNA translation and compete with miRNAs to indirectly influence the expression of coding genes in animals and plants [12, 41]. We predicted two co-expression networks based on lncRNAs and mRNAs have the same miRNA binding sites. The genes were constructed into pCAMBIA2300-35S-OCS vector, then the various combinations was performed transient expression in *Nicotiana benthamiana*. We demonstrated that *ZmmiR159* could cut the target gene *Zm00001d015521* and inhibit its expression level. Moreover, we found *LNC_002923* could inhibit the cleavage of *Zm00001d015521* gene guided by *ZmmiR159c.* The homologs of *Zm00001d015521* in Arabidopsis was found that related to the development and growth of root and shoot, as well as abiotic stress such as drought and nutrition [39], meanwhile, *miR159* has been reported to be involved in abiotic stress response-related pathways and plant growth and development [32, 35]. Based on these results, we concluded that the relatively high abundance of *LNC_002923* could effectively inhibit *ZmmiR159c* to cleave potential coding genes, and further regulated *Zm00001d015521* to play an important regulatory role in response to low nitrogen stress in maize.

### GWAS and RNA-Seq for low nitrogen tolerance

Here it was necessary to combine multiple methods to obtain reliable information. An integrated method comprising GWAS, RNA-Seq, and genomic selection combined with phenotypic data collected from different environments found 16 loci that were significantly associated with soybean resistance to white mold in the field and 11 loci in the greenhouse [42]. In maize, metabolite profiling and GWAS were combined to analyze the mechanisms of response to low-Pi stress, and validation in a recombinant inbred line population found some candidate genes related to yield [20]. We screened 40 consistently LN-responsive candidate genes by integrating RNA-Seq profiles and GWAS data. Notably, among these 12 significant SNPs associated with five root traits, there were seven SNPs associated with Forks, Crossings, Tips, PRL, and CRN located on chromosome 4. A previous GWAS study [43] also found candidate genes controlling root growth and development on chromosome 4. Furthermore, some studies also detected that QTLs related to nitrogen utilization and phosphorus absorption were located on chromosome 4 [44, 45]. The consistency of the results from the present study with previous findings indicates that candidate genes potentially influencing root growth and nutrient utilization under LN stress are probably located on chromosome 4.

### Targets analysis reveals the potential regulatory of LN-responsive lncRNAs in maize

We performed GO and KEGG analyses of lncRNAs and found there were significant numbers of GO terms related to the histidine biosynthetic process, nitrate metabolic process, and chromatin remodeling. Our findings showed that these lncRNAs are involved in the regulation of abiotic stress and nutrition metabolism [12, 46]. Furthermore, KEGG analysis focusing on the biological processes related to plant growth and nutrition metabolism showed that these lncRNAs could interact with mRNAs in the maize seedling in response to LN stress, and were particularly associated with photosynthesis and secondary metabolite synthesis (*p* < 0.05). Taken together, we suggested these lncRNAs participated in the key biological processes including development, biosynthesis, and abiotic stress response.

In the lncRNA-mRNA pairs, 44 and 310 lncRNAs were predicted to regulate transcriptional activation and expression of 24 neighboring and 16 distant genes in *cis* and *trans* manners, respectively, and to be regulated by multiple lncRNAs. In particular, the lncRNAs associated with *Zm00001d051804*, *Zm00001d048998*, *Zm00001d051666*, and *Zm00001d049380*, which could play large roles in determining root growth and responding to the availability of nitrogen in a *cis* and *trans* manner, warrant further study. Our results are interesting for further exploration of the functions of lncRNAs and their target genes in seedlings under LN stress. However, to date, no research has been performed combining GWAS and lncRNAs together to study the mechanism of LN response in maize seedlings.

## Conclusion

We identified several hundred differentially expressed lncRNAs in maize seedlings and used them to construct two co-expression networks that lncRNA as ceRNA based on the “ceRNA hypothesis.” Combining GWAS and expression profiles, a total of 40 consistently LN-responsive candidate genes and lncRNAs potentially related to root traits were identified. Further research on biological function and regulation, including the identification of nearby and distant targets, GO enrichment, and KEGG analysis, should provide useful information for obtaining a deeper understanding of the mechanisms of lncRNA regulation during the early stages of N deficiency in maize seedling development, and for providing new insights enabling increased efficiency of breeding for nitrogen utilization.

## Methods

### Plant materials

Seeds of maize inbred line P178 and *Nicotiana benthamiana* were used in this study (both provided by the Maize Research Institute of Sichuan Agricultural University). The association mapping panel comprised 362 inbred lines with great variation in genetic background obtained from the Southwest China Breeding Program, the population has been reported in our pervious study, including the population structure, genetic diversity, and linkage disequilibrium decay distance [47].

### Growth conditions

Seeds of maize inbred line 178 were surface**–**sterilized with 6% sodium hypochlorite for 15 min, washed twice with deionized water, immersed in saturated CaSO_4_ for 12 h, and then germinated in coarse quartz sand until two leaves were visible. After the endosperms had been removed, the seedlings were placed in a 25-L bucket containing improved half-concentration Hoagland’s nutrient solution for 2 days to adapt to the hydroponic environment and then supplied with full-concentration Hoagland’s nutrient solution. For HN treatment, the nutrient solution consisted of (mM): 4 KNO_3_, 4 MgSO_4_, 5 KCL, 5 CaCL_2_, 1 KH_2_PO_4_, 0.1 Fe-EDTA, 0.046 H_3_BO_4_, 0.009 MnSO_4_, 0.0007 ZnSO_4_, 0.0003 CuSO_4_, and 0.0002 (NH_4_)_6_Mo_7_O_24_. For LN treatment, the 4 mM KNO_3_ was replaced by 0.04 mM KNO_3_. The pH of the solution was adjusted to 6.0–6.5 and nutrient solution was renewed every 2 days. The seedlings were grown in a greenhouse with a photoperiod of 16/8 h (light/darkness) and 25/22 °C, with light intensity of 200 μmol photons m^− 2^ s^− 1^. The relative humidity was maintained at 65%. Three independent replications were conducted and each experiment included at least three seedlings under both HN and LN conditions. The second fully expanded leaf from top to bottom and whole roots were sampled, dried on blotting paper, frozen immediately in liquid N_2_ and finally stored in − 80 °C.

### RNA isolation, and library preparation for lncRNA and small RNA sequencing

Total RNAs were extracted from 14-day-old seedlings of leaves and roots under HN and LN conditions. The libraries of lncRNAs and small RNAs were sequenced on an Illumina Hiseq2500 platform from 12 samples labeled as HN_178L1, HN_178L2, HN_178L3, HN_178R1, HN_178R2, HN_178R3, LN_178L1, LN_178L2, LN_178L3, LN_178R1, LN_178R2, and LN_178R3. RNA purity was checked using the NanoPhotometer® spectrophotometer (IMPLEN, CA, USA). RNA concentration was measured using Qubit® RNA Assay Kit in a Qubit® 2.0 Flurometer (Life Technologies, CA, USA) and integrity was assessed using the RNA Nano 6000 Assay Kit of the Bioanalyzer 2100 system (Agilent Technologies, CA, USA). A total of 3 μg of RNA per sample was used as input material for the RNA and small RNA libraries. After RNA products had been purified (AMPure XP system), library quality was assessed on the Agilent Bioanalyzer 2100 system. The libraries were sequenced on an Illumina Hiseq 2500 platform by Novogene (Beijing, China).

### Bioinformatics identification of lncRNAs

The pipeline used for the identification of lncRNAs is described in Fig. 1a. Clean data were obtained by removing reads containing adapters, reads containing poly-N, and low-quality reads from the raw data. The clean data of high quality that mapped to the B73 RefGen_V4 genome were downloaded directly from genome website (http://www.gramene.org/). An index of the reference genome was built using Bowtie v2.0.6 [48] and paired-end clean reads were aligned to the reference genome using TopHat [49] v2.0.9. After the alignment, the mapped reads of each sample were assembled by both Scripture (beta2) [50] and Cufflinks (v2.1.1) [51] in a reference-based approach. Both methods use spliced reads to determine exon connectivity, but with two different approaches. Next, transcripts predicted to have coding potential by CPC [52] and Pfam Scan [53] were filtered out, and those without coding potential were used as our candidate set of lncRNAs. Cuffdiff (v2.1.1) [51] was used to calculate FPKMs of both lncRNAs and coding genes in each sample. Cuffdiff provides statistical routines for determining differential expression in digital transcript or gene expression data using a model based on the negative binomial distribution. Transcripts with an adjusted *P* < 0.05 were assigned as differentially expressed.

### Analysis of small RNA sequencing data

After the removal of the reads containing poly-N, with 5′ adapter contamination, without 3′ adapter or insert tag, and low-quality reads from the raw data, the small RNA tags were mapped to the maize B73 RefGen_V4 genome by Bowtie [48] without mismatch for further analysis. Rfam 11.0 [54] was applied to remove tags originating from protein-coding genes, repeat sequences, rRNA, tRNA, snRNA, and snoRNA. Then, mapped small RNA tags were used to look for known miRNAs. MiRBase20.0 was used as a reference, while modified software mirdeep2 [55] and sRNA-tools-cli were used to obtain potential miRNAs and draw the secondary structures. The characteristic hairpin structure of miRNA precursors could be used to predict novel miRNAs. The software miREvo [56] and mirdeep2 [55] were integrated to predict novel miRNAs through exploring the secondary structure, the dicer cleavage site, and the minimum free energy of the small RNA tags unannotated in the previous steps. The expression levels were estimated by transcripts per million (TPM) through the following criteria [57]. Differential expression analysis of two conditions/groups was performed using the DESeq R package (1.8.3). The *P*-values were adjusted using the Benjamini & Hochberg method. A corrected *P*-value of < 0.05 was set as the threshold for significant differential expression by default.

### Prediction of target genes

The *cis*-acting mechanism of lncRNAs involves them acting on neighboring target genes. To identify such lncRNAs, a search was performed for coding genes 10–100 kb upstream and downstream of the lncRNAs; the function of those identified was then analyzed. The *trans*-acting mechanism of lncRNAs to identify each other by the expression level. We clustered the genes from different samples to search for common expression modules and then analyzed their function through functional enrichment analysis. Predicting the target genes of miRNA was performed by psRobot_tar in psRobot [58] for plants.

### Validation of quantitative real-time PCR

Quantitative real-time PCR (qRT-PCR) was applied to validate the results of sequencing. Total RNA was extracted from the leaves and roots of line 178 harvested after 0 h, 1 h, 6 h, 12 h, 24 h, 2 days, 4 days, 6 days, 8 days, 10 days, 12 days, and 14 days of nitrogen treatment using Trizol reagent (Invitrogen). Reverse transcription of mRNA and small RNA was performed with PrimeScript™ II 1st Strand cDNA Synthesis Kit (TAKARA) and SYBR® PrimeScript™ miRNA RT-PCR Kit (TAKARA), following the manufacturer’s instructions, respectively.

The qRT-PCR validation of the lncRNAs, mRNAs, and miRNAs was performed with the Roche Cobas Z480 system using FastStart Essential DNA Green Master (Roche) and SYBR® PrimeScriptTM miRNA RT-PCR Kit (TAKARA). Data were analyzed by relative quantification using the myosin (mRNA and lncRNA qPCR) and U6 (miRNA qPCR) genes as standards. Three independent experiments were performed, and each experiment was performed in three technical replicates.

### Construction of co-expression networks related to the development of maize

Based on the “ceRNA hypothesis”, MREs could be acted as the core by which transcripts could influence to each other to regulate their expression levels. First, the target genes of differentially expressed lncRNA and differential expressed mRNAs were combined to analysis. When the targets of lncRNAs were also significantly different, the mRNAs were more likely to be regulated by lncRNAs. Then, filtering out those lncRNAs that could be miRNA precursors based on the homology of lncRNA and miRNA precursors, the software psRobot was applied to predict the target lncRNAs of miRNAs. Third, the upregulated and downregulated results of each group of differentially expressed genes to identify candidate miRNAs that act on mRNAs. Finally, co-expression networks were constructed in which lncRNA was the decoy, miRNA was the core, and mRNA was the target gene.

### Marker data

The MaizeSNP50 BeadChip containing 56,110 SNPs was used for genotyping the panel. Detailed information about this chip can be downloaded from the Illumina MaizeSNP50 website (http://support.illumina.com/array/array_kits/ maizesnp50_dna_analysis_kit/downloads.html) and the positional information of SNPs according to B73 RefGen_v2 can be downloaded from the National Center for Biotechnology Information (NCBI) GEO website.

### Phenotypic measurement

A paper roll growth method was applied for culturing maize [59]. The growth conditions and nutrient solution were the same as previously described. Then, the seeds were surface–sterilized with 6% sodium hypochlorite sodium, and placed on moist filter paper to germinate in the dark. After 2 days, six germinated maize kernels were placed on a double layer of brown germination roll paper (Anchor Paper, St. Paul, MN, USA) that had been pre-moisturized with fungicide solution Captan (2.5 g/L). Germination paper rolls were placed vertically in a 10-l plastic bucket containing 5 l of nutrient solution (HN and LN). The pH of the solution was adjusted to 6.0–6.5. The nutrient solution was renewed every 2 days.

Each paper roll with six seedlings was regarded as an experimental unit. The association line was cultured in a completely random design in three independent replications completed in a greenhouse. After 14 days, seedlings were removed from the plastic bucket and phenotypic traits were measured (three similar seedlings out of six within each roll were sampled, to eliminate possible outliers within lines, and all traits’ means were taken). If measurement could not be performed on a particular day, the nutrient solution was replaced with 30% ethanol to prevent further growth. The root traits were recorded by the WinRhizo program. After measurements had been completed, shoots and roots were collected separately and dried for at least 48 h at 80 °C in an oven dryer.

### Phenotypic analysis

Phenotypic descriptive statistics and correlation coefficients were analyzed using R software. Analyses of variance of seedling traits and broad-sense heritability (H^2^) were performed by SAS. The traits of low nitrogen tolerance index (LNTI) and means were employed to perform GWAS, in which LNTI is the relative trait value that the HN treatment was divided by the same trait value for the LN treatment.

### Association analysis of low nitrogen tolerance-related traits

The allelic frequencies of 255 maize inbred lines and population structure were calculated using the software PowerMarker3.25 [60] and STRUCTURE 2.3 [61], respectively. Kinship was measured with the Genome Association and Prediction Integrated Tool-R package (GAPIT) and STRUCTURE was set to K = 2, in accordance with the results of a previous study [47]. SNPs with low minor allele frequency (MAF) < 0.01 and missing rate > 0.2 were removed, which left 46,108 high-quality SNPs for further association analysis. All markers were evenly distributed on 1–10 chromosomes. The software TASSEL 5.0 [62] was selected to conduct GWAS with the 46,108 high-quality SNPs (MAF > 0.01) using a mixed linear model (MLM) and markers with *P* > 4.6 were considered for candidate gene analysis.

### Transient expression in *Nicotiana benthamiana*

The full-length cDNA of genes and lncRNA were amplified with the specific primers. The precursor of *ZmMIR159* and *ZmMIR164* were amplified from genomic DNA. As a negative control, the sequence of *LNC_002923*, *LNC_003272*, *ZmMIR159* and *ZmMIR164* were conducted to generate site-directed mutagenesis with the Mut Express II Fast Mutagenesis Kit V2 (Vazyme, Nanjing, CN). All the amplified fragments were cloned into the pCAMBIA2300-35S-OCS vector using the *Sal*I restriction site by In-Fusion (TAKARA). The expression plasmids were transformed into *A. tumefaciens* EHA105 and were injected into the epidermis of *N. benthamiana* for transient expression. Four independent experiments were performed. The RNA was extracted from leaves after 2 day using Trizol reagent (Invitrogen) as described above.

## Supplementary Information

**Additional file 1: Fig. S1.** The volcano plot of differentially expressed mRNAs (a, b), TUCPs (c, d) and miRNAs (e, f) between HN and LN conditions in leaf and root. **Fig. S2.** The TPM (Transcripts per million) (a) and length distribution of 18- to 30-nt small RNAs (b). The concentrated length distribution with the peak at 24-nt in the 12 libraries respectively. **Fig. S3.** Expression profiles of mRNA and miRNA during seedling under HN and LN conditions. Cluster heat map of all mRNAs (a) and miRNAs (c) expression in leaf and root. VEEN analysis of differentially expressed mRNAs (b) and miRNAs (d). **Fig. S4.** Characteristics of lncRNAs identified in maize seedling. **Fig. S5.** Functional analysis of the LN-responsive lncRNAs. The enriched Kyoto Encyclopedia of Genes (KEGG) pathways in leaf (a, c) and root (b, d). **Fig. S6.** Fourty-five and 232 consistent candidate genes were detected in root and leaf under LN condition (a) and LNTI (b) by combing with RNA-Seq and GWAS.

**Additional file 2: Table S1.** Known miRNAs and novel miRNAs identified from RNA-Seq.**Table S2.** The targets of lncRNAs predicted by co-location and co-expression. **Table S3.** LncRNA-mRNA pairs of *cis*-acting. **Table S4.** LncRNA-mRNA pairs of *trans*-acting. **Table S5.** The GO analysis of lncRNAs. **Table S6.** The KEGG analysis of lncRNAs. **Table S7.** The candidate genes identified by GWAS and RNA-Seq. **Table S8.** Pearson (r) correlations between all 17 traits. **Table S9.** The targets of lncRNAs contained significant SNPs for multiple root traits. **Table S10.** The information of primer sequences used in this study. **Table S11.** Trait abbreviation and description collected by manually and WinRizo.

## Data Availability

All original data can be downloaded from the NCBI database (accession number: PRJNA661965 and PRJNA662035).

## Abbreviations

HN: High nitrogen
LN: Low nitrogen
GWAS: Genome-wide association study
SNPs: Single-nucleotide polymorphisms
LncRNAs: Long-noncoding RNAs
CeRNAs: Competing endogenous RNAs
MREs: MicroRNA response elements
LD: Linkage disequilibrium
TPM: Transcripts per million
qRT-PCR: Quantitative real-time PCR
H^2^: Heritability
LNTI: Low nitrogen tolerance index
MAF: Minor allele frequency
MLM: Mixed linear model
TUCPs: Transcripts of uncertain coding potential
SRN: Seminal root number
RDW: Root dry weight
SL: Shoot length
TRL: Total root length
CRN: Crown root number
