# Supplementary figures and images for: Transcriptomic and genome-wide association study reveal long noncoding RNAs responding to nitrogen deficiency in maize
Source: BMC Plant Biol. 2021 Feb 12;21:93. doi: 10.1186/s12870-021-02847-4 (PMC7879672; doi:10.1186/s12870-021-02847-4)

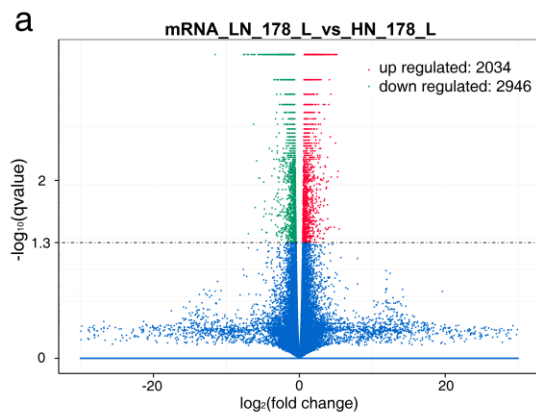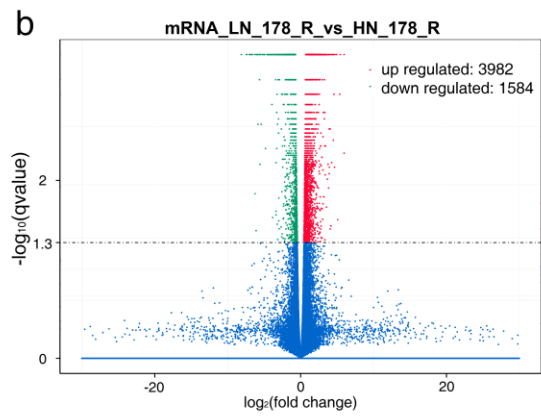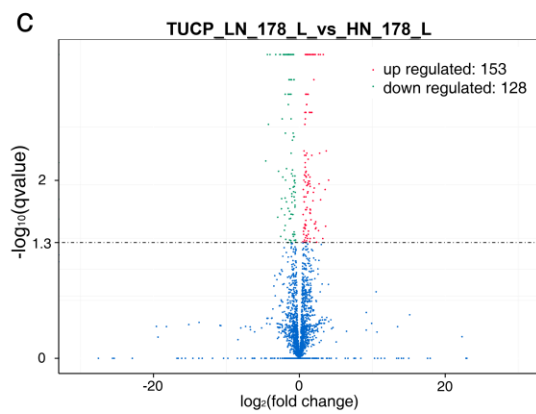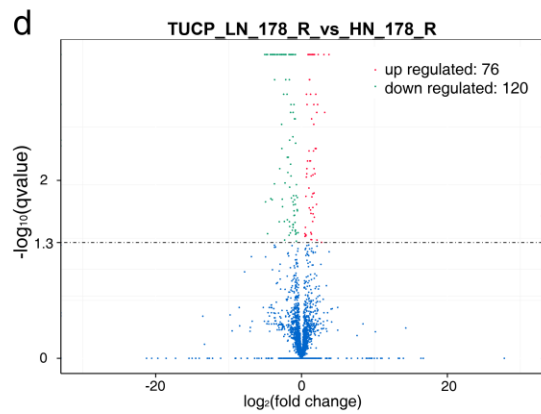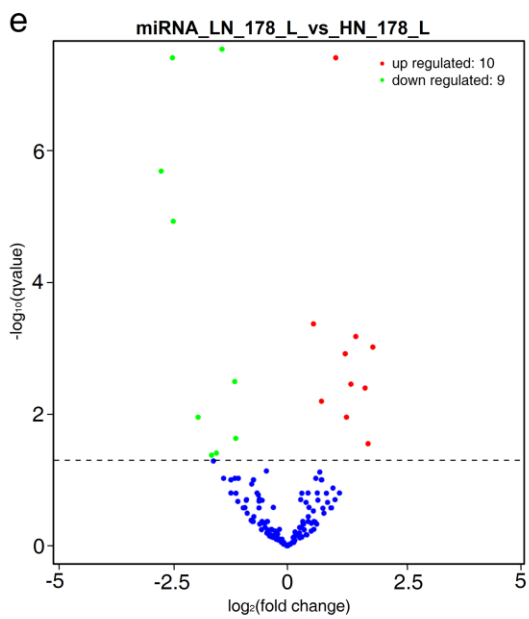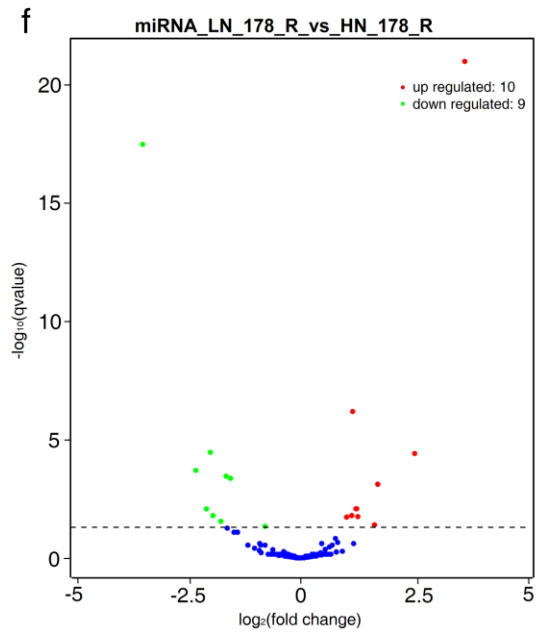

Supplement: Supplementary file 1 — Additional file 1: Fig. S1. The volcano plot of differentially expressed mRNAs (a, b), TUCPs (c, d) and miRNAs (e, f) between HN and LN conditions in leaf and root. Fig. S2. The TPM (Transcripts per million) (a) and length distribution of 18- to 30-nt small RNAs (b). The concentrated length distribution with the peak at 24-nt in the 12 libraries respectively. Fig. S3. Expression profiles of mRNA and miRNA during seedling under HN and LN conditions. Cluster heat map of all mRNAs (a) and miRNAs (c) expression in leaf and root. VEEN analysis of differentially expressed mRNAs (b) and miRNAs (d). Fig. S4. Characteristics of lncRNAs identified in maize seedling. Fig. S5. Functional analysis of the LN-responsive lncRNAs. The enriched Kyoto Encyclopedia of Genes (KEGG) pathways in leaf (a, c) and root (b, d). Fig. S6. Fourty-five and 232 consistent candidate genes were detected in root and leaf under LN condition (a) and LNTI (b) by combing with RNA-Seq and GWAS. [file 12870_2021_2847_MOESM1_ESM.zip › Figure S1.pdf]

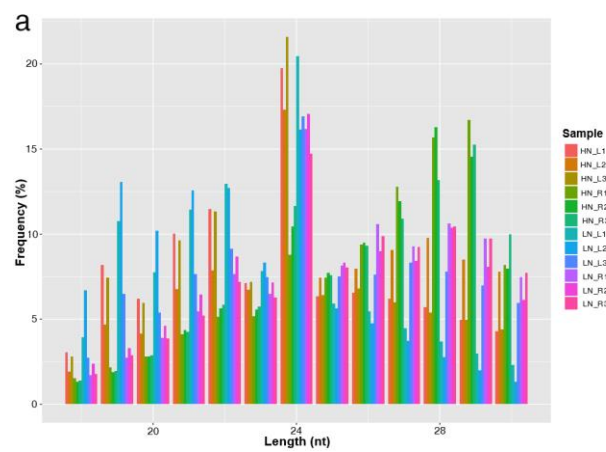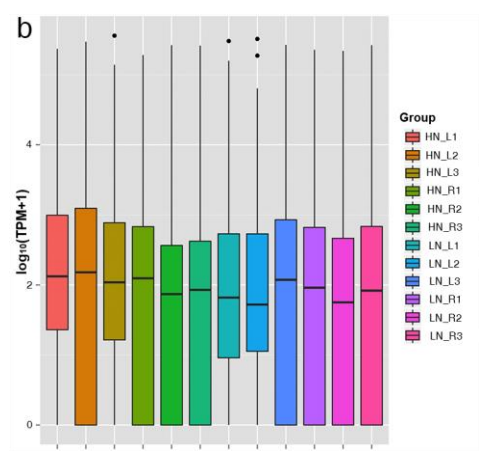

Supplement: Supplementary file 1 — Additional file 1: Fig. S1. The volcano plot of differentially expressed mRNAs (a, b), TUCPs (c, d) and miRNAs (e, f) between HN and LN conditions in leaf and root. Fig. S2. The TPM (Transcripts per million) (a) and length distribution of 18- to 30-nt small RNAs (b). The concentrated length distribution with the peak at 24-nt in the 12 libraries respectively. Fig. S3. Expression profiles of mRNA and miRNA during seedling under HN and LN conditions. Cluster heat map of all mRNAs (a) and miRNAs (c) expression in leaf and root. VEEN analysis of differentially expressed mRNAs (b) and miRNAs (d). Fig. S4. Characteristics of lncRNAs identified in maize seedling. Fig. S5. Functional analysis of the LN-responsive lncRNAs. The enriched Kyoto Encyclopedia of Genes (KEGG) pathways in leaf (a, c) and root (b, d). Fig. S6. Fourty-five and 232 consistent candidate genes were detected in root and leaf under LN condition (a) and LNTI (b) by combing with RNA-Seq and GWAS. [file 12870_2021_2847_MOESM1_ESM.zip › Figure S2.pdf]

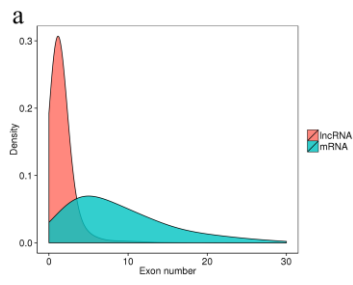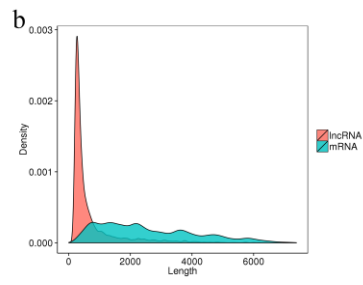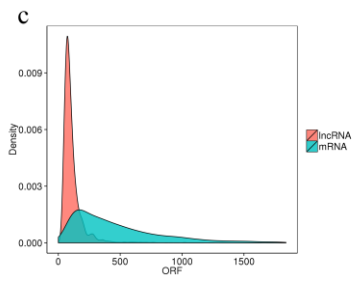

Supplement: Supplementary file 1 — Additional file 1: Fig. S1. The volcano plot of differentially expressed mRNAs (a, b), TUCPs (c, d) and miRNAs (e, f) between HN and LN conditions in leaf and root. Fig. S2. The TPM (Transcripts per million) (a) and length distribution of 18- to 30-nt small RNAs (b). The concentrated length distribution with the peak at 24-nt in the 12 libraries respectively. Fig. S3. Expression profiles of mRNA and miRNA during seedling under HN and LN conditions. Cluster heat map of all mRNAs (a) and miRNAs (c) expression in leaf and root. VEEN analysis of differentially expressed mRNAs (b) and miRNAs (d). Fig. S4. Characteristics of lncRNAs identified in maize seedling. Fig. S5. Functional analysis of the LN-responsive lncRNAs. The enriched Kyoto Encyclopedia of Genes (KEGG) pathways in leaf (a, c) and root (b, d). Fig. S6. Fourty-five and 232 consistent candidate genes were detected in root and leaf under LN condition (a) and LNTI (b) by combing with RNA-Seq and GWAS. [file 12870_2021_2847_MOESM1_ESM.zip › Figure S4.pdf]

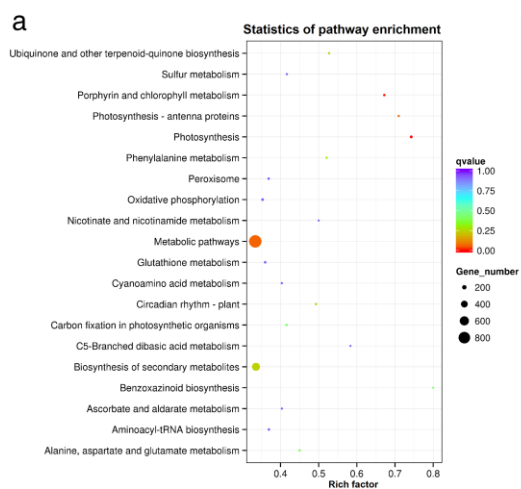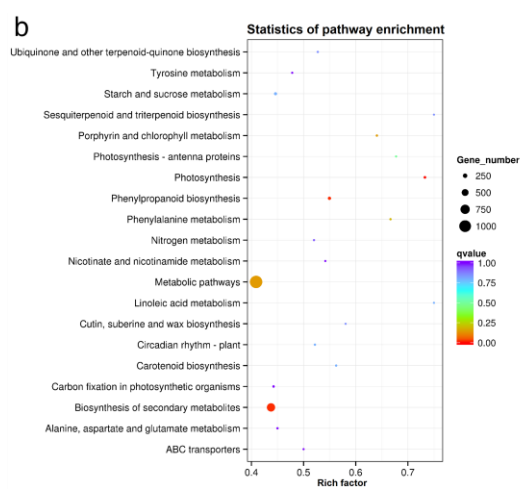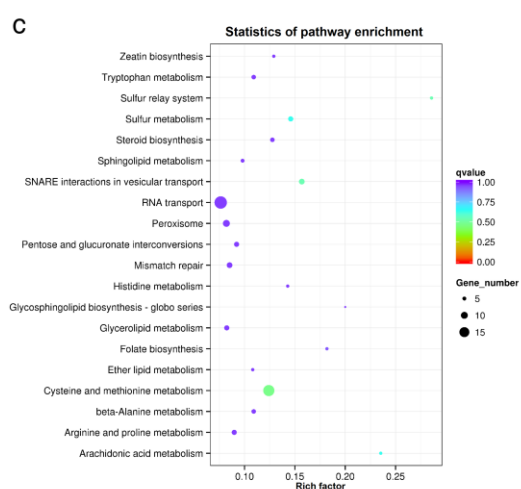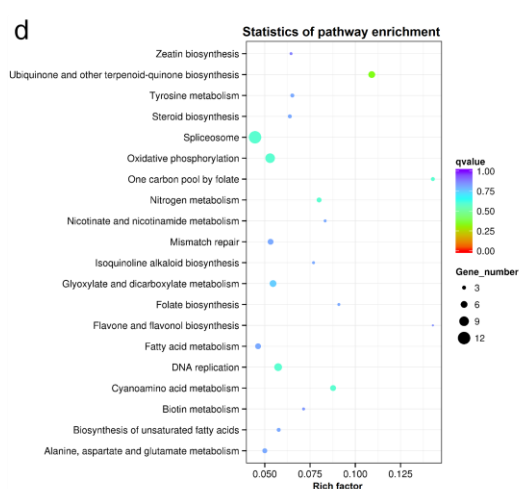

Supplement: Supplementary file 1 — Additional file 1: Fig. S1. The volcano plot of differentially expressed mRNAs (a, b), TUCPs (c, d) and miRNAs (e, f) between HN and LN conditions in leaf and root. Fig. S2. The TPM (Transcripts per million) (a) and length distribution of 18- to 30-nt small RNAs (b). The concentrated length distribution with the peak at 24-nt in the 12 libraries respectively. Fig. S3. Expression profiles of mRNA and miRNA during seedling under HN and LN conditions. Cluster heat map of all mRNAs (a) and miRNAs (c) expression in leaf and root. VEEN analysis of differentially expressed mRNAs (b) and miRNAs (d). Fig. S4. Characteristics of lncRNAs identified in maize seedling. Fig. S5. Functional analysis of the LN-responsive lncRNAs. The enriched Kyoto Encyclopedia of Genes (KEGG) pathways in leaf (a, c) and root (b, d). Fig. S6. Fourty-five and 232 consistent candidate genes were detected in root and leaf under LN condition (a) and LNTI (b) by combing with RNA-Seq and GWAS. [file 12870_2021_2847_MOESM1_ESM.zip › Figure S5.pdf]

**a**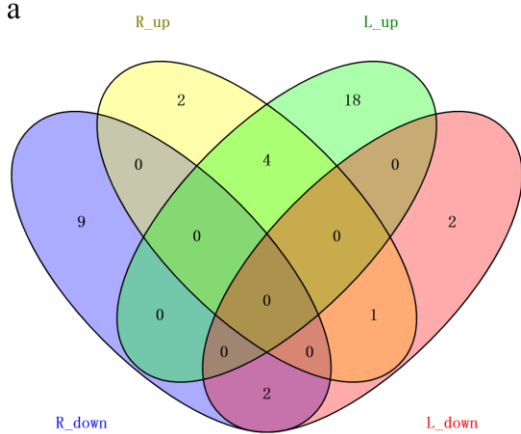**b**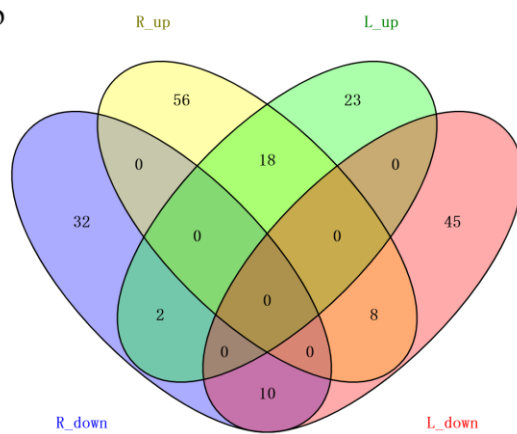

Supplement: Supplementary file 1 — Additional file 1: Fig. S1. The volcano plot of differentially expressed mRNAs (a, b), TUCPs (c, d) and miRNAs (e, f) between HN and LN conditions in leaf and root. Fig. S2. The TPM (Transcripts per million) (a) and length distribution of 18- to 30-nt small RNAs (b). The concentrated length distribution with the peak at 24-nt in the 12 libraries respectively. Fig. S3. Expression profiles of mRNA and miRNA during seedling under HN and LN conditions. Cluster heat map of all mRNAs (a) and miRNAs (c) expression in leaf and root. VEEN analysis of differentially expressed mRNAs (b) and miRNAs (d). Fig. S4. Characteristics of lncRNAs identified in maize seedling. Fig. S5. Functional analysis of the LN-responsive lncRNAs. The enriched Kyoto Encyclopedia of Genes (KEGG) pathways in leaf (a, c) and root (b, d). Fig. S6. Fourty-five and 232 consistent candidate genes were detected in root and leaf under LN condition (a) and LNTI (b) by combing with RNA-Seq and GWAS. [file 12870_2021_2847_MOESM1_ESM.zip › Figure S6.pdf]
