# Supplementary material for: Transcriptomic and genome-wide association study reveal long noncoding RNAs responding to nitrogen deficiency in maize
Source: BMC Plant Biol. 2021 Feb 12;21:93. doi: 10.1186/s12870-021-02847-4 (PMC7879672; doi:10.1186/s12870-021-02847-4)
Supplement: Supplementary file 1 — Additional file 1: Fig. S1. The volcano plot of differentially expressed mRNAs (a, b), TUCPs (c, d) and miRNAs (e, f) between HN and LN conditions in leaf and root. Fig. S2. The TPM (Transcripts per million) (a) and length distribution of 18- to 30-nt small RNAs (b). The concentrated length distribution with the peak at 24-nt in the 12 libraries respectively. Fig. S3. Expression profiles of mRNA and miRNA during seedling under HN and LN conditions. Cluster heat map of all mRNAs (a) and miRNAs (c) expression in leaf and root. VEEN analysis of differentially expressed mRNAs (b) and miRNAs (d). Fig. S4. Characteristics of lncRNAs identified in maize seedling. Fig. S5. Functional analysis of the LN-responsive lncRNAs. The enriched Kyoto Encyclopedia of Genes (KEGG) pathways in leaf (a, c) and root (b, d). Fig. S6. Fourty-five and 232 consistent candidate genes were detected in root and leaf under LN condition (a) and LNTI (b) by combing with RNA-Seq and GWAS. [file Figure S3.pdf › Figure S3.pdf]

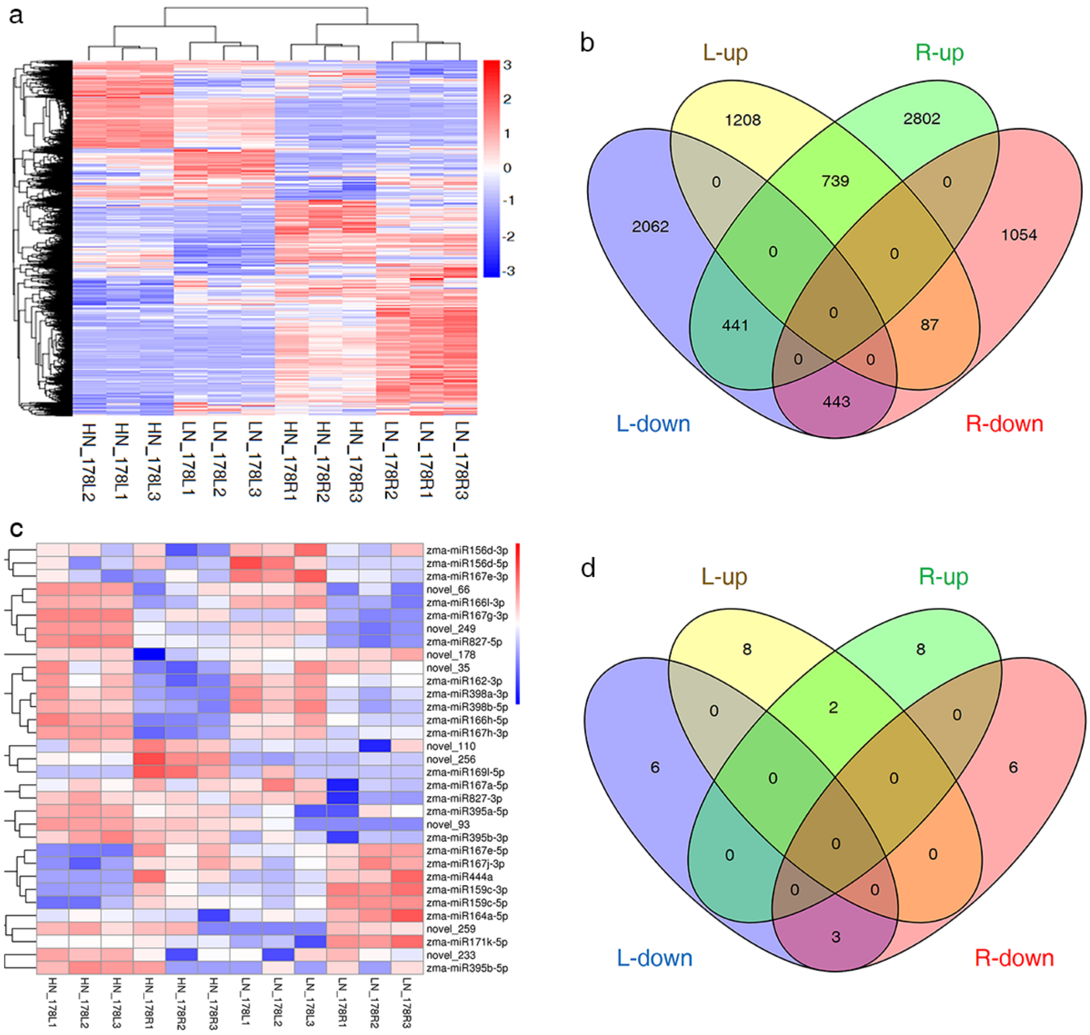

a
3
2
1
0
-1
-2
-3
HN_178L2
HN_178L1
HN_178L3
LN_178L1
LN_178L2
LN_178L3
HN_178R1
HN_178R2
HN_178R3
LN_178R2
LN_178R1
LN_178R3
b
L-up
R-up
L-down
R-down
1208
2802
0
739
0
2062
1054
0
0
0
441
87
0
0
443
c
zma-miR156d-3p
zma-miR156d-5p
zma-miR167e-3p
novel_66
zma-miR166l-3p
zma-miR167g-3p
novel_249
zma-miR827-5p
novel_178
novel_35
zma-miR162-3p
zma-miR398a-3p
zma-miR398b-5p
zma-miR166h-5p
zma-miR167h-3p
novel_110
novel_256
zma-miR169l-5p
zma-miR167a-5p
zma-miR827-3p
zma-miR395a-5p
novel_93
zma-miR395b-3p
zma-miR167e-5p
zma-miR167j-3p
zma-miR444a
zma-miR159c-3p
zma-miR159c-5p
zma-miR164a-5p
novel_259
zma-miR171k-5p
novel_233
zma-miR395b-5p
HN_178L1
HN_178L2
HN_178L3
HN_178R1
HN_178R2
HN_178R3
LN_178L1
LN_178L2
LN_178L3
LN_178R1
LN_178R2
LN_178R3
d
L-up
R-up
L-down
R-down
8
8
0
2
0
6
6
0
0
0
0
0
0
0
3
